# Genetic and phylogenetic analysis of dissimilatory iodate-reducing bacteria identifies potential niches across the world’s oceans

**DOI:** 10.1038/s41396-021-01034-5

**Published:** 2021-07-02

**Authors:** Victor Reyes-Umana, Zachary Henning, Kristina Lee, Tyler P. Barnum, John D. Coates

**Affiliations:** Department of Plant and Microbial Biology, University of California, Berkeley, CA USA

**Keywords:** Biogeochemistry, Biogeochemistry, Microbial ecology

## Abstract

Iodine is oxidized and reduced as part of a biogeochemical cycle that is especially pronounced in the oceans, where the element naturally concentrates. The use of oxidized iodine in the form of iodate (IO_3_^−^) as an electron acceptor by microorganisms is poorly understood. Here, we outline genetic, physiological, and ecological models for dissimilatory IO_3_^−^ reduction to iodide (I^−^) by a novel estuarine bacterium, *Denitromonas* sp. IR-12. Our results show that dissimilatory iodate reduction (DIR) by strain IR-12 is molybdenum-dependent and requires an IO_3_^−^ reductase (*idrA*) and likely other genes in a mobile cluster with a conserved association across known and predicted DIR microorganisms (DIRM). Based on genetic and physiological data, we propose a model where three molecules of IO_3_^−^ are likely reduced to three molecules of hypoiodous acid (HIO), which rapidly disproportionate into one molecule of IO_3_^−^ and two molecules of iodide (I^−^), in a respiratory pathway that provides an energy yield equivalent to that of nitrate or perchlorate respiration. Consistent with the ecological niche expected of such a metabolism, *idrA* is enriched in the metagenome sequence databases of marine sites with a specific biogeochemical signature (high concentrations of nitrate and phosphate) and diminished oxygen. Taken together, these data suggest that DIRM help explain the disequilibrium of the IO_3_^−^:I^−^ concentration ratio above oxygen-minimum zones and support a widespread iodine redox cycle mediated by microbiology.

## Introduction

Iodine (as ^127^I) is the heaviest stable element of biological importance and an essential component of the human diet due to its role in thyroxine biosynthesis in vertebrates [1–3]. Iodine is enriched in marine environments where it exists in several oxidation states, reaching concentrations of up to 450 nM [4]. In these environments, organisms such as kelp bioconcentrate iodine as iodide (I^−^) and produce volatile iodine species such as methyl iodide [5]. These volatile iodine species contribute to the destruction of tropospheric ozone (a major greenhouse gas) and aerosol formation at the marine boundary layer, consequently resulting in cloud formation and other local climatic effects [1, 6]. Despite the global biological and geochemical importance of iodine, little is known about its biogeochemistry in the ocean [4]. For instance, the biological mechanism accounting for the unexpected chemical disequilibrium between I^−^ and iodate (IO_3_^−^) in seawater (I^−^:IO_3_^−^ disequilibrium) remains unknown [4]. At the physicochemical conditions of seawater, iodine is most stable as IO_3_^−^ [7], yet measurements of IO_3_^−^ and I^−^ in regions with high biological productivity (e.g., marine photic zones, kelp forests, or sediments), reveal an enrichment of the I^−^ ion beyond what can be explained through abiotic reduction [7, 8] with ferrous iron [9] or sulfide.

Among numerous explanations proposed for I^−^ enrichment, microbial IO_3_^−^ reduction is particularly compelling. The high reduction potential (IO_3_^−^/I^−^ *E*_*h*_ = 0.72 V at pH 8.1) [7, 10] makes IO_3_^−^ an ideal electron acceptor for microbial metabolism in marine environments. Early studies indicated common microorganisms such as *Escherichia coli* and *Shewanella putrefaciens*, reduce IO_3_^−^ to I^−^ [10, 11]. Subsequent studies associated this metabolism with the inadvertent activity of DMSO respiratory reductase enzymes in marine environments, along with specific enzymes (i.e., perchlorate reductase, nitrate reductase) that reduce IO_3_^−^ in vitro [10, 12, 13]. However, there is little evidence that organisms hosting these enzymes are capable of growth by IO_3_^−^ reduction. While inadvertent IO_3_^-^ reduction might be mediated by marine bacteria possessing DMSO reductases, until recently, no definitive evidence existed that global IO_3_^−^ reduction is a microbially assisted phenomenon.

In support of a microbial role for the observed I^−^:IO_3_^−^ disequilibrium, previous studies demonstrated that at least one member each of the common marine genera *Pseudomonas* and *Shewanella* are capable of IO_3_^−^ reduction [13–15]. More recently, IO_3_^−^ reduction by *Pseudomonas* sp. strain SCT was associated with a molybdopterin oxidoreductase closely related to arsenite oxidase [15]. As part of this work, a dedicated biochemical pathway was proposed involving two peroxidases associated with a heterodimeric IO_3_^−^ reductase (Idr) [15]. The putative model proposes a four-electron transfer mediated by Idr, resulting in the production of hydrogen peroxide and hypoiodous acid [15]. Two peroxidases detoxify the hydrogen peroxide while a chlorite dismutase (Cld) homolog dismutates the hypoiodous acid into I^−^ and molecular oxygen, which is subsequently reduced by the organism [15]. The proposed pathway involving a molecular O_2_ intermediate is analogous to canonical microbial perchlorate respiration [16]. By contrast, Toporek et al. [17]. using the IO_3_^−^ respiring *Shewanella oneidensis* demonstrated the involvement of an unidentified reductase associated with the *mtrAB* multiheme cytochrome, suggesting an alternative dissimilatory iodate reduction (DIR) pathway. The disparate mechanisms underscore the potential diversity of IO_3_^−^ respiratory processes. As such, identification of additional DIR microorganisms (DIRM) would clarify which genes are required for this metabolism and enable identification of IO_3_^−^ respiratory genes in metagenomes.

With this as a primary objective, we identified a novel marine DIRM, *Denitromonas* sp. strain IR-12, that obtained energy for growth by coupling IO_3_^−^ reduction to acetate oxidation. Taxonomic analysis placed this organism in the *Denitromonas* genus commonly associated with marine environments [18]. We used comparative genomics to identify the core genes involved in IO_3_^−^ respiration, which formed a distinct mobile genomic island. Reverse genetics, physiology, and comparative genomic data were used to propose a new model for DIR, with a confirmed role for a molybdopterin-dependent IO_3_^−^ reductase (IdrAB) [15]. A phylogenetic analysis was used to establish the distribution of this metabolism across the tree of life and measure the degree to which the genomic island is subject to horizontal gene transfer. Finally, metagenomic analysis identified the *idrA* gene in the Tara oceans datasets, enabling the correlation of DIR populations with ocean chemistry. These results together enabled the proposed model for the global distribution of the DIR metabolism and the ecology of the microorganisms involved.

## Results and discussion

### Isolation of *Denitromonas* sp. IR-12

**Fig. 1:**
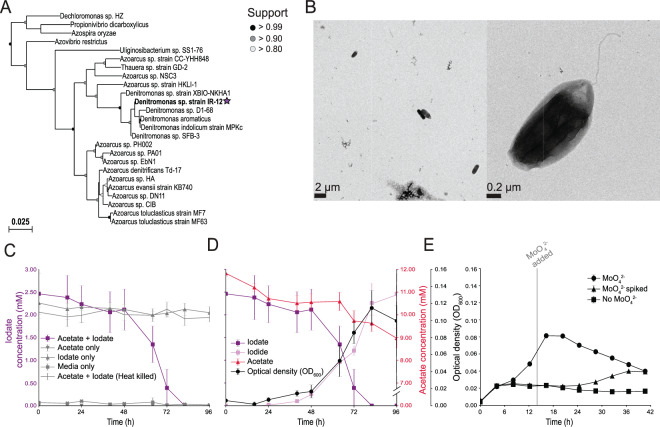
Phylogeny and physiology of *Denitromonas* sp. IR-12. **A** 16S rRNA gene phylogeny of *Denitromonas* sp. IR-12 (denoted by a purple star) belonging to a subclade of *Azoarcus*, separate from other known *Azoarcus* species. **B** TEM images of an active culture of *Denitromonas* sp. IR-12 with the scale at 2 μm (left) and 0.2 μm (right) taken on a Technai 12 TEM. **C** Iodate consumption across all five conditions assessed in the growth experiment in **D**. *N* = 3 and error bars show standard deviation. **D** Iodate consumption (), acetate consumption (), iodide production (), and growth (; measured as optical density at λ=600 nm; OD600) in an active culture of *Denitromonas* sp. IR-12 growing anaerobically. *N* = 3 and error bars show standard deviation. **E** Optical density (OD600) in the presence (), absence (), and amendment of MoO42- after 14 hours incubation (). *N* = 7 and error bars show standard deviation.

Strain IR-12 was obtained from estuarine sediment samples by selective enrichment under anoxic conditions followed by single colony isolation on aerobic agar plates. Analysis of the 16S rRNA indicated an axenic culture composed of a single phylotype belonging to the *Denitromonas* genus in the class *Betaproteobacteria* identical to an uncultured *Denitromonas* clone from a metagenomic sample (GenBank: KF500791.1) (Fig. 1A). The closest cultured relatives were *D. indolicum* strain MPKc [19] (GenBank: AY972852.1, 99.46% similarity) and *D. aromaticus* (GenBank: AB049763.1, 99.40% similarity). Strain IR-12 is a facultative anaerobe with rod-shaped motile cells 1–2 μm long and 0.5 μm diameter containing a single polar flagellum (Fig. 1B).

### Physiology and energetics of *Denitromonas* sp. IR-12

Cells of *Denitromonas* sp. IR-12 grew on basal medium with acetate and IO_3_^−^ as the sole electron donor and acceptor, respectively (Fig. 1C, D). Ion chromatography and growth studies revealed that IO_3_^−^ was quantitatively reduced to I^−^ with concomitant cell density increase. No growth or acetate consumption occurred in the absence of IO_3_^−^. Similarly, no IO_3_^−^ reduction occurred in the absence of acetate or in heat killed controls. These results indicated that IO_3_^−^ reduction was enzymatically mediated coupled to acetate oxidation and growth. Acetate-free control cultures reduced micromolar amounts of IO_3_^−^ (114 ± 34 µM, mean ± standard deviation, *n* = 3) which was attributable to residual acetate carried over from the inoculum (Fig. 1C). *Denitromonas* sp. IR-12 consumed 2.46 ± 0.499 mM IO_3_^−^ (mean ± standard deviation, *n* = 3) while oxidizing 2.86 ± 0.427 mM acetate (mean ± standard deviation, *n* = 3) with a final optical density (OD_600_) increase of 0.109. This is equivalent to an average stoichiometry of 0.86 mol IO_3_^−^ per mol acetate. The doubling time of cells grown under these conditions is 10.96 h (*µ* = 0.06) which is roughly three times longer than cells growing under aerobic conditions in analogous media (3.42 h, *µ* = 0.20). The morphological consistency between *Denitromonas* sp. IR-12 and *E. coli*, suggests that an OD_600_ increase of 1.0 is equivalent to 0.39 grams of cell dry weight per liter [20] and that ~50% of cell dry weight is comprised of carbon [21]. Using these numbers, the corrected stoichiometry accounting for acetate incorporation into cell mass is 93% of the theoretical value according to:

3 CH_3_COOH + 4 IO_3_^−^ → 6 CO_2_ + 4 I^−^ + 6 H_2_O

Our calculations indicate that 30.72% of total carbon is assimilated into biomass while the remaining is respired. Such a result is typical for highly oxidized electron acceptors such as oxygen, nitrate, or perchlorate [16, 22]. In support of this, the calculated Gibb’s free energy for the reduction of IO_3_^−^ per mole of electrons transferred during iodate respiration on acetate is −97.44 kJ/mol e^−^ (assuming pH of 8.1, T = 298.15 K, and 1 atm) [23]. These values place the energy provided through IO_3_^−^ respiration akin to that of perchlorate respiration (ClO_4_^−^/Cl^−^, *E*^*o*^′ = +0.797 V) [16], and between that of aerobic respiration (O_2_/H_2_O, *E*^*o*^′ = +0.820 V) and nitrate reduction (NO_3_^−^/N_2_, *E*^*o*^′ = +0.713 V) [24]. This suggests a similar degree of carbon assimilation would be expected for IO_3_^−^ respiration [22].

### DIR is molybdate dependent

The reduction of oxyanions like IO_3_^−^, such as bromate, chlorate, perchlorate, and nitrate, is typically catalyzed by enzymes belonging to the DMSO reductase superfamily of molybdopterin oxidoreductases [25]. These enzymes require molybdenum as a cofactor in order to donate two electrons at a time to the receiving molecule [26]. To determine if phenotypic IO_3_^−^ reduction was molybdenum-dependent, we passaged *Denitromonas* sp. IR-12 six times in aerobic, molybdate-free minimal media to remove any trace molybdenum as described in Chaudhuri et al. [27]. As expected, and similarly to observations with perchlorate-reducing microorganisms [27], omitting molybdenum from the oxic medium did not affect the aerobic growth of *Denitromonas* sp. IR-12 (data not shown). In contrast, no growth or IO_3_^−^ reduction was observed when these cells were passaged into molybdenum-free anoxic media with IO_3_^−^ as the electron acceptor (Fig. 1E). When 0.1 mM sodium molybdate was added into the non-active cultures at 14 h post inoculation, growth and IO_3_^−^ resumed (Fig. 1E). These results demonstrate that IO_3_^−^ respiration by *Denitromonas* sp. IR-12 is molybdenum dependent and are consistent with the involvement of a DMSO oxidoreductase in IO_3_^−^ reduction [27].

### Core genes required for DIR

**Fig. 2:**
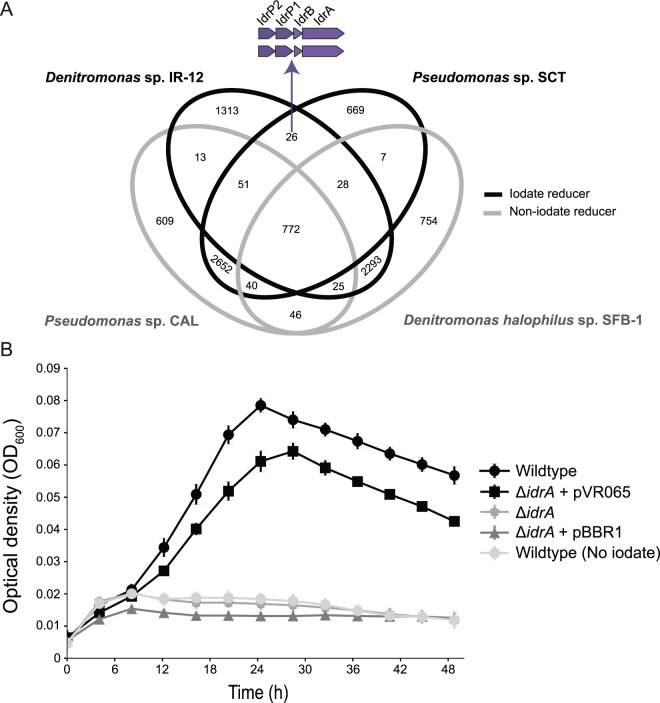
Identification of a unique gene cluster in iodate reducing genomes enabling the identification and characterization of the iodate reductase (IdrA). **A** A four-way comparison between two genomes from confirmed DIRM (black line) and two genomes from closely related non-DIRM (gray line) identifying 26 shared genes among the two taxonomically distinct iodate reducing bacteria (see Table S2). The four genes involved in DIR are shown above the Venn diagram in purple. **B** Anaerobic growth of wildtype of Denitromonas sp. IR-12 in the presence () or absence () of iodate is shown in comparison to the idrA mutant (), the idrA mutant complemented with an empty vector (), or with idrA complemented in trans (). *N* = 8 and error bars represent standard deviation.

To identify the genes required for IO_3_^−^ respiration we performed a comparative genomic analysis between the genomes of the IO_3_^−^ respiring species (*Denitromonas* sp. IR-12 and *Pseudomonas* sp. SCT), and the non-IO_3_^−^ respiring close relatives (*D. halophilus* SFB-1, and *Pseudomonas* sp. CAL). Additionally, *Pseudomonas* and *Denitromonas* are from phylogenetically distinct classes (*Gammaproteobacteria* and *Betaproteobacteria*, respectively), reducing the likelihood of shared gene content [28]. We surmised that DIRM must share a unique gene (or set of genes) that enables IO_3_^−^ reduction. This comparison identified 26 genes uniquely shared by the two DIRM and not found in the closely related non-IO_3_^−^ respiring species (Fig. 2A; Table S2). Four of these genes were present in a gene cluster that contained genes for alpha and beta subunits of a DMSO reductase family molybdopterin enzyme related to arsenite oxidase (AioAB) [29] supporting our result of a molybdenum dependency for this metabolism. The remaining two genes in the cluster were closely related to cytochrome C peroxidases *ccp1* and *ccp2*, possibly involved electron shuttling and oxidative stress responses [30, 31]. These four genes were similar to those identified by Yamazaki et al. under the proposed nomenclature *idrA*, *idrB*, *idrP*_*1*_, *idrP*_*2*_ for *Pseudomonas* sp. SCT [15] (Fig. 2B). A SignalP analysis showed that *idrP*_*1*_ and *idrP*_*2*_ possessed a signal sequence for periplasmic secretion via the Sec pathway, while *idrB* used the Tat pathway [32]. By contrast *idrA* did not have a signal peptide sequence, suggesting its protein product is co-transported with IdrB into the periplasm [33]. Based on this evidence, we concluded that dissimilatory IO_3_^−^ reduction in *Denitromonas* sp. IR-12 occurs entirely in the periplasm, consistent with the observation by Amachi et al. that associated IO_3_^−^ reductase activity in the periplasmic fractions of *Pseudomonas* strain SCT [14]. Notably, the gene cluster lacked a quinone oxidoreductase suggesting that *Denitromonas* sp. IR-12 involves the expression of a non-dedicated quinone oxidoreductase.

Evidence associating IdrAB to DIR, currently relies on the IO_3_^−^ consuming activity of crude cell extracts of *Pseudomonas* strain SCT and differential expression of *idrABP*_*1*_*P*_*2*_ under IO_3_^−^ reducing conditions [15]. To validate the association between these genes and DIR in *Denitromonas* sp. IR-12, we developed a genetic system to perform targeted knockouts (see Table S1 and  Supplementary Methods for details). The *idrA* gene was targeted since its associated molybdenum cofactor ultimately mediates the reduction of the oxyanion [25]. Upon introduction of an in-frame deletion at the *idrA* locus, the organism was incapable of growth via IO_3_^−^ respiration (Fig. 2B) while growth under oxic conditions remained unimpaired. Complementation of *idrA* on a low copy number vector (pVR065) restored the IO_3_^−^ respiring phenotype demonstrating that the *idrA* gene is a prerequisite to enable IO_3_^-^ respiration (Fig. 2B). Our identification of a second DIRM, in addition to *Pseudomonas* strain SCT, with an IdrAB suggests that IO_3_^−^ reduction requires a specialized molybdopterin oxidoreductase, and that other molybdopterin oxidoreductases in the genome cannot rescue the phenotype. Furthermore, our work demonstrates a distinct difference from IO_3_^−^ reduction by the multiheme cytochrome associated reductase in *Shewanella* and suggests that the ability to reduce IO_3_^−^ may have evolved at least twice independently.

### An alternative DIR model

The current model for IO_3_^−^ respiration by *Pseudomonas* strain SCT proposes the donation of electrons from the quinone pool via a cytochrome c to IdrAB, to initiate reduction of IO_3_^−^ to HIO and H_2_O_2_. H_2_O_2_ is reduced to H_2_O by the peroxidases IdrP_1_ and IdrP_2_, while a chlorite dismutase (Cld)-like enzyme converts HIO to I^−^ and ½O_2_, a catalytic function that has never been demonstrated for Cld or Cld-like proteins [15]. The resultant oxygen is then further respired to H_2_O by a terminal oxygen reductase. The putative participation of a Cld-like protein was based on expression data rather than empirically determined activity [15]. Furthermore, comparative genomics does not support the general involvement of Cld in IO_3_^−^ respiration, as *cld* is never co-located with the iodate reduction gene cluster and is notably absent from all but two of the 145 putative DIRM genomes identified in NCBI GenBank (see below) including the genome of *Denitromonas* sp. IR-12.

**Fig. 3:**
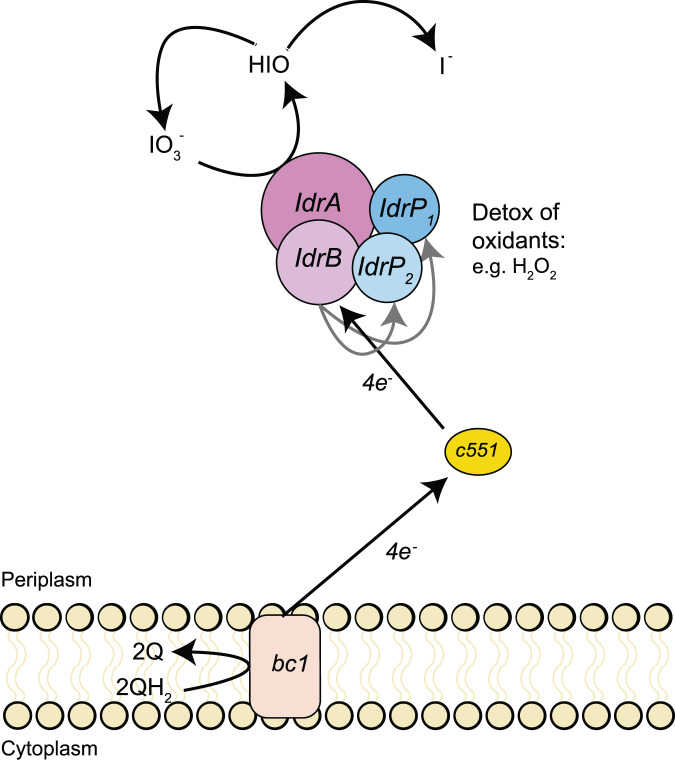
Mechanistic model of iodate reduction. A representation of the electron flow (black arrows) from the quinone pool to iodate in *Denitromonas sp.* IR-12. QH_2_ reduced quinone, Q oxidized quinone, bc1 bc1 complex, IO_3_ iodate, HIO hypoiodous acid, I iodide, H_2_O_2_ hydrogen peroxide. Gray arrows represent micromolar production of yet unknown oxidant that is detoxified by IdrP_1_ and IdrP_2_.

Since *Denitromonas* sp. IR-12 genome lacks *cld*-like genes, we propose that the primary mechanism of IO_3_^−^ respiration by this organism relies on the complex and reactive chemistry of iodine oxyanions [34] and that the peroxidases IdrP_1_ and IdrP_2_ serve a critical detoxification role for inadvertent oxidants generated rather than being central components of the pathway itself. In the *Denitromonas* sp. IR-12 model (Fig. 3A), IdrAB accepts electrons from cytochrome c551, and performs a four-electron transfer, similarly to the mechanism of perchlorate reductase (Pcr) [12], with a resultant production of the chemically unstable intermediate hypoiodous acid (HIO). This intermediate then undergoes abiotic disproportionation to yield I^−^ and IO_3_^−^ in a 2:1 ratio as reported in alkaline aquatic environments [35, 36], and is simplistically represented by the following equation:

3 HIO → 2 I^−^ + IO_3_^−^ + 3 H^+^

The resultant IO_3_^−^ subsequently cycles back into the reductive pathway. In this manner, the cell completes the 6-electron reduction of IO_3_^−^ to I^−^ without invoking a Cld-like enzyme with putative capacity to dismutate IO^−^ to I^−^ and O_2_. This model is similar to the cryptic model for some species of perchlorate-reducing microorganism which rely on the chemical reactivity of the unstable pathway intermediate chlorite (ClO_2_^−^) with reduced species of iron or sulfur to prevent toxic inhibition [12, 37]. We propose that the initial reduction of IO_3_^−^ at the IdrA inadvertently produces low levels of incidental toxic H_2_O_2_. This is analogous to the production of hypochlorite (ClO^−^) by respiratory perchlorate-reducing microorganisms during respiration of perchlorate or chlorate [38, 39]. To protect themselves from this reactive chlorine species, perchlorate respiring organisms have evolved a detoxifying mechanism based on redox cycling of a sacrificial methionine rich peptide [39]. In the *Denitromonas* sp. IR-12 model for IO_3_^−^ respiration the cytochrome c peroxidases play the critical detoxification role against inadvertent H_2_O_2_ production, rather than a central role for the reductive pathway as proposed for *Pseudomonas* strain SCT [15] (Fig. 3A). Such a model is not only parsimonious with the predicted biochemistries and abiotic reactivities of the proteins and iodine oxyanions involved but is also consistent with the micromolar quantities of H_2_O_2_ observed by Yamazaki et al. during the reduction of millimolar quantities of IO_3_^−^ by *Pseudomonas* strain SCT [15].

### Evolutionary history of DIR

**Fig. 4:**
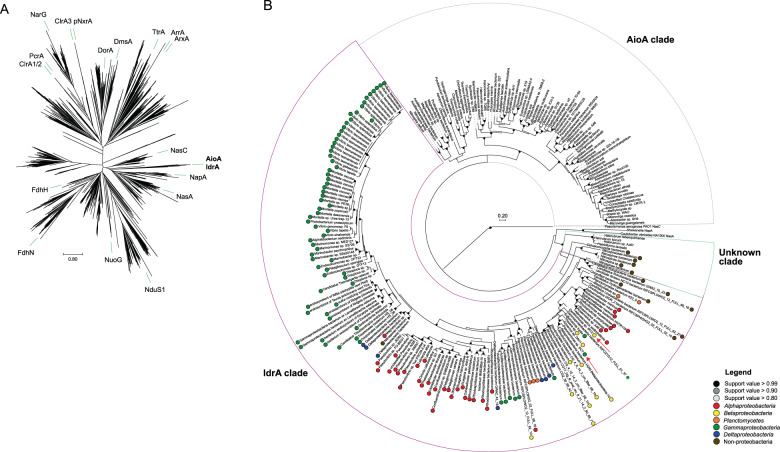
Phylogeny and taxonomic distribution of IdrA. **A** Phylogeny of molybdopterin oxidoreductases (Pfam 00384) using pre-aligned proteins from the representative proteomes 55 dataset. Green bars indicate location of an individual protein in each branch belonging to the labeled group. **B** Phylogeny of the iodate reductase (IdrA; purple), arsenite oxidase (AioA; gray), and an unknown clade (light green) that contains proteins from organisms showing demonstrated arsenite oxidation abilities. Colored circles along the edges of the IdrA clade indicate the different class each organism belongs to. Red arrows indicate the location of IdrA from the two confirmed iodate-reducing microorganisms.

**Fig. 5:**
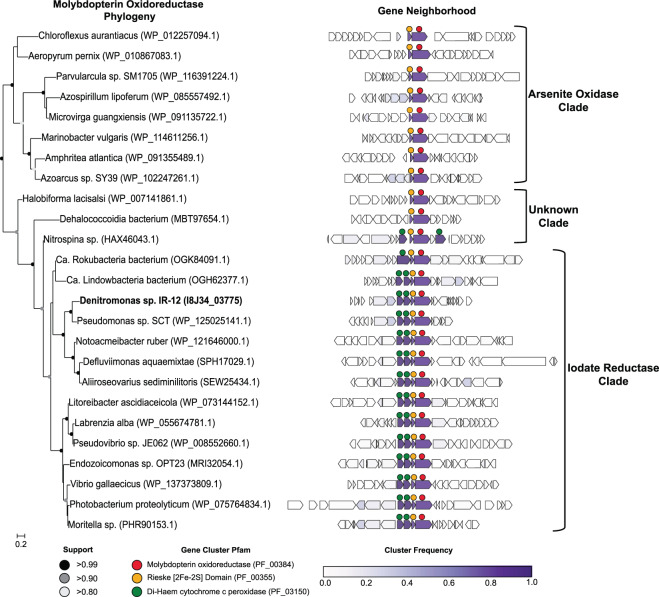
Phylogeny and gene neighborhoods of arsenite oxidase, iodate reductase, and the associated unknown clade. A pruned tree of the molybdopterin oxidoreductase phylogeny (left) showing a representative subset of genomes identified from Fig. 4B. *Denitromonas* sp. IR-12 is illustrated in bold and locus tags are provided in parentheses. Gene neighborhoods (right) show ten genes upstream and downstream (if present) from the *idrA* locus. Individual genes were clustered into groups based on amino acid similarity using MMSeqs2 and the frequency of genomes possessing an individual cluster is colored by the intensity of purple. Circles above each gene represents either the molybdopterin oxidoreductase (), the associated Rieske containing subunit (), or the di-haem cytochrome c peroxidase protein families ().

Core genes for DIR were used to define the phylogenetic distribution of this metabolism. Numerous homologs, some showing between 50 and 80% amino acid identity to the catalytic subunit of IdrA, were identified among genomes in NCBI GenBank. A phylogenetic tree of the DMSO reductase family (Fig. 4A, B) confirms previous results indicating that arsenite oxidase alpha subunit (AioA) is the most closely related characterized enzyme to IdrA [15]. The extent of the IdrA clade was difficult to define because IdrA from *Denitromonas* sp. IR-12 and *Pseudomonas* sp. SCT are closely related. To determine whether more IdrA homologs in this clade function as IO_3_^−^ reductases or arsenite oxidases, we performed a gene neighborhood analysis looking at the ten genes both upstream and downstream of either the *idrA* or *aioA* locus and clustered them using MMseqs2 [40] (Figs. 5,  S1). We observed a clear distinction in neighborhood synteny between genes mostly closely to *idrA* versus those most closely related to *aioA*. All neighborhoods in the *idrA* clade showed conserved synteny at *idrABP*_*1*_*P*_*2*_ (Fig. 5), whereas organisms with an AioA, showed an alternative gene structure, notably missing the cytochrome c peroxidases. Based on this pattern, all organisms possessing *idrABP*_*1*_*P*_*2*_ genes are likely DIRM. The outgroups of IO_3_^−^ reductase in this phylogeny are homologs found in *Halorubrum* spp., which are known to oxidize arsenite [41], and a *Dehalococcodia* bacterium (GCA_002730485.1), which also lacks the cytochrome c peroxidases in its gene neighborhood (Figs. 5,  S1). Further research into these proteins may provide more information on the transition from arsenite oxidase to IO_3_^−^ reductase.

Genes mediating IO_3_^−^ reduction were identified in 145 genomes from bacteria in the *Alphaproteobacteria*, *Betaproteobacteria*, and *Gammaproteobacteria*. Deeper branching members included members of *Planctomycetaceae* and several others belonging to the Candidate Phyla Radiation group such as, *Ca. Rokubacteria*, *Ca. Lindowbacteria*, and NC10 (Fig. 4B) [42–44]. DIR seemed most prevalent in the phylum *Proteobacteria*, which is a pattern that has been observed for some other rare metabolisms [45]. The discordance between the taxonomy of the host organisms and the phylogeny of IdrA (Figs. 4B, S2) [46] suggested that DIR is a horizontally transferred metabolism. For example, IdrA in the *Gammaproteobacterium Pseudomonas* sp. SCT was most closely related to IdrA in *Betaproteobacteria* such as *Azoarcus* sp. DN11. Additional evidence for horizontal gene transfer in individual genomes included insertion sites at the 3′ end of tRNAs, a skew in GC content, and association with other horizontally transferred genes [47, 48]. In *Denitromonas* sp. IR-12, there was no significant GC skew or direct inverted repeats. However, we observed a tRNA^Gly^ roughly 72 kbp downstream of the *idrABP*_*1*_*P*_*2*_ locus which was previously demonstrated to be an integration site by Larbig et al. in *P. stutzeri* [49]. Additionally, numerous heavy metal resistance markers, like *mer* and *cus* genes, were found near the *idrABP*_*1*_*P*_*2*_ locus (1.2 and 22 kbp away respectively), further suggesting horizontal transfer [47, 50, 51]. A method to detect genomic islands in complete genomes predicted the *idrABP*_*1*_*P*_*2*_ locus to be its own 5.8 kbp genomic island in *Azoarcus* sp. DN11, which has a complete genome and a closely related IdrA [52]. Therefore, while there is poor conservation of genes surrounding *idrABP*_*1*_*P*_*2*_ and questions remain about its recent evolution, the high degree of conservation of *idrABP*_*1*_*P*_*2*_ locus itself and the phylogenetic pattern of inheritance support its description as an iodate reduction genomic island (IRI) that is subject to horizontal gene transfer. In addition to the perchlorate reduction genomic island (PRI) [45] the IRI represents one of the few respiratory genomic islands known that crosses large phylogenetic boundaries (class, order, and family).

### Distribution of DIR populations in global oceans

**Fig. 6:**
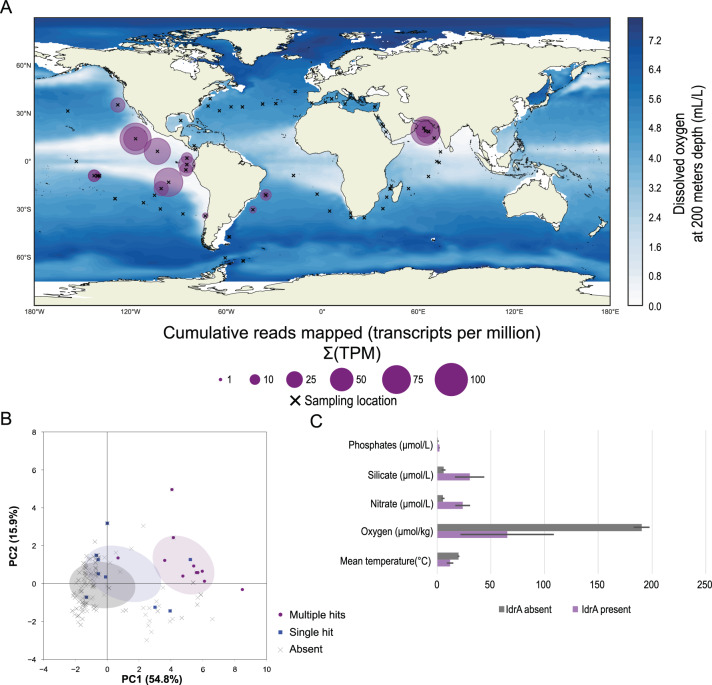
Analysis of Tara Oceans dataset identifies possible ecological niche above oxygen-minimum zones. **A** A map indicating sampled locations during the Tara expedition (x) alongside sampling locations with IdrA present (purple circles). Markers overlaid directly on top of each other demonstrate transect samples from different depths at a given location. Size of purple circle shows the cumulative TPM at a particular site. **B** A principal component analysis displaying the first two principal components. Locations are grouped by IdrA absent (x), presence of a single IdrA hit (), or presence of multiple hits (). Ellipses represent 1 standard deviation of the mean. The color of the ellipse corresponds to the variable grouping. **C** The means of select environmental variables at IdrA present sites (purple) and IdrA absent sites (gray). Error bars indicate 95% confidence interval. Units for each of the variables are located by the variable name.

Many of the organisms with genes for DIR were identified in diverse marine habitats where IO_3_^−^ reduction is suspected to occur (Table S3). For example, *Litorimicrobium taeanense* is an aerobic, non-motile, *Alphaproteobacterium* isolated from a sandy beach in Taean, South Korea [53]. Other organisms such as *Endozoicomonas* sp. OPT23 and *Litoreibacter ascidiaceicola* were isolated from marine animals such as the intertidal marine sponge (*Ophlitaspongia papilla*) and the sea squirt (*Halocynthia aurantium*), respectively [54, 55]. Additionally, organisms known to accumulate iodine, such as algae [56] are associated with these bacteria as is the case with the bacterium *Rhodophyticola porphyridii* and the red algae *Porphyridium marinum* [57]. To investigate this marine prevalence further we used the *idrA* subunit as a marker gene to determine DIRM distribution across the Tara Oceans metagenome dataset. Our approach also identified the read abundance mapping to these unique IdrA hits at the different sites by using the transcripts per million (TPM) method for read quantification [58, 59]. With this method, the number of unique IdrA hits was directly proportional to the number of reads mapped to the hits (Figs. 6A, S3). In general, locations with few unique IdrA hits lacked reads mapping to IdrA (Fig. S3). We observed that 77% (74/96) of the hits arose from the mesopelagic zone at an average depth of about 461 meters (range 270–800 m) across identified stations (Fig. S4). The remaining hits arose predominantly in epipelagic zones, such as the deep chlorophyll maximum (DCM) in 21% of cases (20/96) and far fewer hits were observed in the mixed layer (1/96) or the surface water layer (1/96).

Although the presence of *idrA* exhibited some variability in depth, a geochemical feature common to all these hits was low-oxygen concentrations. The vast majority of hits mapped to well-documented oxygen minimal zones in the Arabian Sea [60, 61] and the Eastern Tropical Pacific [62–64]. Similarly, the North Pacific Subtropical and Polar Front (MRGID:21484) and the North Pacific Equatorial Countercurrent provinces (MRGID:21488) are two Longhurst provinces with OMZs that stand out in the Western hemisphere. At each of these locations, the median dissolved oxygen concentration at *idrA* positive locations was consistently lower than the dissolved oxygen concentrations at *idrA* absent locations (65.24 µmol/kg versus 190.41 µmol/kg; Fig. 6C). Among locations containing more than one *idrA* hit, the average oxygen concentration was about six times lower (11.03 µmol/kg); however, this average was skewed upward due to one outlier condition with 18 *idrA* hits (Cumulative TPM of 89.30; Fig. S4) occurring at a dissolved oxygen concentration of 95.4 µmol/kg (TARA_137_DCM_0.22–3). Environments meeting these conditions were the most common in mesopelagic zones broadly. One notable exception were the multiple hits at the DCM at station 137. However, further inspection of this particular DCM revealed that the sample matched the high nitrate and phosphate concentrations and low dissolved oxygen of other *idrA* positive mesopelagic environments more closely than the comparatively more oxygenated surface waters or deep chlorophyll maxima. Research from Farrenkopf et al. indicated that bacteria are responsible for IO_3_^−^ reduction in oxygen-minimum zones [13, 65]. Further, Saunders et al. showed a preferential expression of *aioA*-like genes in the Eastern Pacific oxygen-minimum zones, which our evidence now suggests are IO_3_^−^-reductases (IdrA) [29].

To test whether locations with *idrA* possessed a unique chemical signature, we ran a principal component analysis using the variables associated with sample environments. Together the first two components of these geochemical variables explained 70.7% of the variance observed between *idrA* present and *idrA* absent samples. We determined that *idrA* presence was correlated most strongly with increased nitrate, phosphate, and silicate concentrations (Figs. 6B, C,  S5). Additionally, *idrA* presence was negatively correlated with dissolved oxygen concentrations (Figs. 6B, C, S5). Such an observation is atypical for highly productive nitrate and phosphate depleted OMZs [60, 66, 67]^,^. A possible explanation for this observation is that DIRM inhabit a unique niche above OMZs where residual O_2_ concentrations above 20 µmol/kg prevents *fnr*-dependent expression of nitrate reductase [68, 69]. Given the range wide range of dissolved O_2_ concentrations with *idrA* genes present (0.70–237.22 µmol/kg; Fig. S4), these organisms potentially use IO_3_^−^ as an alternative electron acceptor in both dysoxic (20–90 µmol/kg) and suboxic zones (≤20 µmol/kg). Furthermore, recent observations from Hardisty et al. show that iodate reduction occurs at locations with average O_2_ concentrations of 11 µmol/kg, providing further evidence of a possible niche above the OMZ core for organisms with *idrA* [70]. Our explanation corroborates results from Farrenkopf and Luther that shows an I^−^ maximum occurring at the boundary of the OMZ [61], but further studies into the biochemistry of IO_3_^−^ reduction under suboxic conditions and the contribution of DIRM to I^−^ formation at this transition zone are necessary to undeniably link the I^-^ maximum with the presence of *idrA* directly.

### Significance

Here we describe a new organism, *Denitromonas* sp. IR-12, that grows by IO_3_^−^ respiration which is mediated by a novel molybdenum-dependent DMSO reductase. The conserved core genes associated with DIR and the chemistry of iodine oxyanions are consistent with a hybrid enzymatic-abiotic pathway by which IdrAB reduces IO_3_^−^ to HIO, which abiotically disproportionates to I^−^ and IO_3_^−^ [35, 36]. In this model, cytochrome c peroxidase like proteins (IdrP_1_ and IdrP_2_) detoxify reactive H_2_O_2_ byproducts. Genes for this metabolism are part of a highly conserved IO_3_^−^ reduction genomic island (IRI). Organisms harboring the IRI belong to phylogenetically distinct taxa, many of which are associated with marine sediments or multicellular hosts, suggesting that DIR is a horizontally transferred metabolism across marine ecosystems over geologic time. The abundance of IdrA genes across ocean metagenomes strongly correlates to oxygen-minimum zones, indicating a niche for this metabolism in low-oxygen, high-nitrate habitats across the ocean, from sediments to oxygen-minimum zones to the surfaces of multicellular organisms. In high-nitrate, low-oxygen conditions, bacteria with the IRI can use IO_3_^−^ as an electron acceptor to obtain energy from the oxidation of organic matter. We propose that IO_3_^−^ is constantly replenished by a combination of the chemical oxidation of I^−^ at the sea surface and by direct and indirect biological iodide oxidation [7, 71, 72]; however, more research into the mechanisms by which IO_3_^−^ is replenished is needed. IO_3_^−^ is typically scarce (0.45 µM in seawater) [4], so DIRM must compete with IO_3_^−^ reduction by chemical reductants (such as ferrous iron [9] or sulfide [73] emerging from deeper anoxic waters) and by inadvertent biological activity, such as by algae, that contribute to the relative depletion of IO_3_^−^ in those waters [7, 61, 65, 74, 75]. By analogy, perchlorate-reducing bacteria, which are common but sparse due to low natural abundance of perchlorate [76], may provide further insight into the ecology of DIRM broadly. The rarity of IO_3_^−^ reduction genes among bacteria despite the ability of the metabolism to be horizontally transferred likely reflects the evolutionary constraints of growth by DIR. Intriguingly, one organism, *Sedimenticola thiotaurini*, seemingly possesses both perchlorate and IO_3_^−^ reduction pathways, presenting future opportunities to study the ecology of these metabolically versatile microorganisms [77]. Moreover, organisms such as *Vibrio* spp. and *Moritella* spp. show some degree of vertical transfer for the IRI throughout recent evolutionary history, indicating possible niches among sea fauna and cold environments where DIR is biogeochemically favorable (Fig. 4B). Future studies addressing the affinity of IdrAB for IO_3_^−^ may also shed light on how DIRM thrive at such low environmental concentrations. Additionally, further research into the chemistry of iodine oxyanions may provide insight on the intermediates of IO_3_^−^ reduction. Addressing these open questions may ultimately shed light on new potential niches for DIRM and provide a role for these organisms in potentiating iodine redox cycling globally.

### Description of *Denitromonas* sp. strain IR-12

*Denitromonas* sp. IR-12 is a facultatively anaerobic chemoorganotroph, gram negative, rod-shaped, 1.5–2.0 µM long by 0.6–0.7 µM wide, and motile by means of a unipolar flagellum (Fig. 1B). Colonies are circular, smooth, and range in color from transparent to an opaque/whitish-sky blue color after 48 h of growth on R2A agar at 30 °C. Extended growth on R2A agar (96 or more hours) results in a light coral pink colony color. *Denitromonas* sp. IR-12 grows by oxidizing D-glucose, lactate, or acetate with concomitant reduction of oxygen (O_2_), nitrate (NO_3_^−^), or iodate (IO_3_^−^). On iodate, *Denitromonas* sp. IR-12 grows optimally at 2 mM IO_3_. It exhibits an IC_50_ of 3.73 mM IO_3_^−^ and 8.67 mM I^−^. Growth occurs between 20 and 30 °C but it is routinely grown at 30 °C. It grows at a range of 0–5% salinity with an optimum growth rate between 1–3% NaCl on minimal media. *Denitromonas* sp. IR-12 has an innate resistance to tetracycline (10 µg/µL) and chloramphenicol (25 µg/µL) but is sensitive to kanamycin, which inhibits growth at concentrations as low as 5 µg/µL.

The genome of *Denitromonas* sp. IR-12 is 5,181,847 bp (average coverage 64.2×) with 4697 CDS, a G + C content of 66.54%, 57 tRNAs, one tmRNA, one CRISPR, and a single plasmid 81,584 bp long whose function remains unclear. The full genome has been deposited in GenBank (BioProject ID PRJNA683738) currently consisting of 202 contigs. An analysis of the genome using KEGG mapper [78] identifies a very versatile carbohydrate and energy metabolism including full pathways for glycolysis, the TCA cycle, the pentose phosphate pathway, and pyruvate oxidation). Phylogenetically, *Denitromonas* sp. IR-12 belongs to the class *Betaproteobacteria*; however, its phylogeny beyond this class becomes less clear. The 16S rRNA locus suggests that *Denitromonas* sp. IR-12 is in a subclade of *Azoarcus*, which belongs to the family *Zoogloeaceae* [79]. However, the NCBI database suggests that the genus *Denitromonas* belongs to the family *Rhodocyclaceae*. Additional studies into the phylogeny of *Denitromonas* spp. are needed to determine the phylogeny of this genus.

Strain IR-12, was enriched from marine sediment from the Berkeley Marina in the San Francisco Bay during the Fall of 2018 (further details explained in methods section). The strain has been deposited in the American Type Culture Collection (Type Strain Deposit Number: TSD-242).

## Methods

### Media, chemicals, and culture conditions

Anaerobic enrichment cultures from marine environments were grown at 30 °C using a minimal media containing the following per liter: 0.54 g NH_4_Cl, 0.14 g KH_2_PO_4_, 0.20 g MgCl_2_・6 H_2_O, 0.14 g Na_2_SO_4_・10 H_2_O, 20.0 g NaCl, 0.24 g Na_2_MoO_4_ 0.20 g, and 2.5 g NaHCO_3_ with an added vitamin mix and mineral mix (composition of mixes in Supplementary Methods). Oxygen was removed from the media and bottles were dispensed in an 80% N_2_/20% CO_2_ atmosphere. Anaerobic subcultures for isolation were grown in Artificial Pore Water (APM) medium at 30 °C (30.8 g NaCl, 1.0 g NH_4_Cl, 0.77 g KCl, 0.1 g KH_2_PO_4_, 0.20 g MgSO4·7H2O, 0.02 g CaCl_2_・2 H_2_O, 7.16 g HEPES, along with vitamin and mineral mixes. A post sterile addition of 34.24 mL 0.4 M CaCl_2_ and 26.07 mL 2 M MgCl_2_・6H_2_O was added to each liter of APM media. Conditions with lactate, acetate, iodate, and nitrate all used the sodium salts of these compounds. Conditions without molybdenum omitted Na_2_MoO_4_ from the mineral mixes. Aerobic cultures were all grown either on APM, R2A (HiMedia, USA), or R2A agar (BD Biosciences, USA). Kanamycin concentrations when used were at one tenth the standard concentrations on plates (5 mg/L, Sigma Aldrich, USA) and at one fourth the standard concentration in liquid (12.5 mg/L). All compounds were purchased through Sigma Aldrich (Sigma Aldrich, USA). Growth of tubes were measured either using the Thermo Scientific GENESYS 20 or the TECAN Sunrise 96-well microplate reader set at a wavelength of 600 nm. For growth measurements in Hungate tubes, a special adapter was built to measure the tubes on the GENESYS 20. Growth experiments using the microplate reader were run in an anerobic glove bag under an atmosphere of 97.8% N_2_ and 2.2% H_2_.

### Strain characterization experiments

Imaging of *Denitromonas* sp. IR-12 was performed on a Technai-12 transmission electron microscope by the staff at the University of California Berkeley Electron Microscope Laboratory. Motility was made by visual observation of a wet mount under a compound microscope at ×100 magnification. Inhibitory concentrations of iodate and iodide were determined by using the fitting a dose response curve over a wide range of iodate/iodide concentrations (in halving concentrations between 0.39 and 200 mM) in APM with 10 mM lactate and using the peak OD_600_ as the response variable. Optimal salinity was measured by calculating the max growth rate during the exponential phase at salinities of 0, 0.125, 0.25, 0.5, 1.0, 2.0, 3.0, 4.0, 5.0, 7.5, and 10%. The GraphPad Prism software suite (version 8.4.0) was used to calculate the IC_50_ values. Temperature range was determined by growth of *Denitromonas* sp. IR-12 on R2A agar plates at 20, 25, and 30 °C. Evaluation of antibiotic resistance was performed by dissolving antibiotics tetracycline (10 µg/µL) and chloramphenicol (25 µg/µL) into R2A agar plates, and streak plating *Denitromonas* sp. IR-12. Kanamycin sensitivity was determined similarly by testing concentrations of 50.0, 25.0, 12.5, and 5.0 µg/µL.

### Isolation of dissimilatory iodate-reducing bacteria

Sediment from the top two inches of a tidal flat in the San Francisco Bay estuary at the Berkeley Marina (37°86′56.4″ N, −122°30′63.9″ W) was added to anaerobic media bottles at 25 g sediment/100 mL for isolation of dissimilatory iodate-reducing bacteria. Samples were degassed and amended with 200 µM iodate for 3 days, and subsequently amended with 10 mM acetate and 2 mM iodate to enable growth of heterotrophic iodate-reducing bacteria. Enrichments that showed iodate reduction to iodide were then passaged at least five times into fresh minimal media with 10 mM acetate and 2 mM iodate. To ensure purity of the passaged enrichment culture, the organism was plated aerobically onto an agar plate containing the minimal media, and a single colony was isolated from this plate.

### Strains and plasmids

All plasmids, primers and strains constructed are listed in Table S1. The *E. coli* strain used for plasmid propagation was XL1-Blue, while WM3064 was used to perform conjugations. Plasmid pNTPS138, a generous gift from the Kathleen Ryan Lab at UC Berkeley, was used for the SacB counterselection. Plasmid pBBR1-MCS2 is a low copy expression vector and was used for complementation experiments. All expression plasmids and deletion vectors were constructed using the Benchling software suite (San Francisco, USA). Plasmids were assembled from genomic DNA either by Gibson assembly or restriction digestion and ligation using standard procedures. Gibson assembly was carried out using NEB HiFi 2x Master Mix, and remaining enzymes and master mixes were ordered from New England Biosciences (NEB, USA). Additional plasmids were built using primers to remove unwanted sequences by site directed mutagenesis and re-circularizing the resulting product with the KLD Enzyme Mix (NEB, USA). Plasmids were routinely isolated using the Qiaprep Spin Miniprep kit (Qiagen, USA), and all primers were ordered from Integrated DNA Technologies (IDT, Coralville, IA). Since most sequences in the iodate reduction cluster contain at minimum 60% GC content, amplification is relatively challenging. Amplification of these challenging portions of the genome were optimized as follows: Amplification of DNA for generating assembly products was performed using Q5 DNA Polymerase 2x Master Mix (NEB, USA) with 3% DMSO. Annealing temperatures for each reaction was determined by subtracting the Tm provided by the NEB Tm calculator (https://tmcalculator.neb.com) for each primer pair by 1.8 °C. All *Denitromonas* sp. IR-12 strains (pre- or post-transformation) were propagated from glycerol stocks (25% glycerol) stored at −80 °C, grown on a plate for up to 72 h, picked and then grown for an additional 48–72 h in liquid R2A. For additional information on plasmid construction, performing transformations, and conjugations in *Denitromonas* sp. IR-12 see Supplementary Methods.

### Iodate and iodide quantification

A Dionex IonPac AS25 Anion Exchange Column was used on an ICS-1500 Ion Chromatography system (Thermo Fischer, USA) exclusively to measure the consumption of iodate and acetate, as well as the production of iodide in all samples. Briefly, all samples are diluted 1:20 in deionized water and loaded onto the autosampler for processing. Standards are made by serial dilution starting with 1 mM of the standard molecule. Iodate and iodide standards were linear across a range of 0.008–1.000 mM (*R*^*2*^ > 0.99). Acetate standards were near linear (*R*^*2*^ > 0.98) between 0.031 and 1.000 mM; however, acetate standards were fit along a quadratic model (*R*^*2*^ > 0.99), as suggested by Brinkmann et al. for quantifying weak acids [80]. All samples were run in triplicate using a flow rate of 1 mL/min and a 36 mM NaOH eluent. Acetate peaks were consistently detected at 3.6 min, iodate peaks were consistently detected at 3.8 min, and iodide peaks were consistently detected at 11.5 min.

### Genome sequencing, comparative genomics, and phylogenetic analysis

Genome sequencing was carried out on an HiSeq4000 using 150 bp paired end reads (Illumina, USA). This work used the Vincent J. Coates Genomics Sequencing Laboratory at UC Berkeley, supported by NIH S10 OD018174 Instrumentation Grant. FastQC 0.11 was used to assess the quality of the illumina reads and sickle 1.33 to trim the reads. The genome was subsequently assembled using SPAdes 3.9 [81] and the assembly graph was assessed for completion using bandage [82]. The Prokka (version 1.14) pipeline was then used to generate the genome annotations and the general feature format file (.gff), which allowed for genome navigation and visualization on the Artemis software (available at http://sanger-pathogens.github.io) [83]. To search for the iodate reduction island, MMseqs2 was used to cluster homologous proteins in the amino acid FASTA (.faa) files from *Denitromonas* sp. IR-12, *P. stutzeri* sp. SCT, *D. halophilus* SFB-1, and *P. stutzeri* sp. CAL by subfamily [40]. A presence and absence matrix for each subfamily was generated and represented as a four-way Venn diagram using pyvenn (https://github.com/tctianchi/pyvenn). To identify additional iodate reductase proteins in public databases, a profile-HMM was constructed using HMMER 3.0 following a multiple sequence alignment using MUSCLE 3.8 on the molybdopterin oxidoreductase (Pfam_00384) seed set and *Denitromonas* sp. IR-12 /*P. stutzeri* SCT IdrA proteins [84, 85]. A separate arsenite oxidase (AioA) profile-HMM was created using analogous methods. Genomes from high probability BLAST hits for IdrA and AioA (*E* value = 0) and from the AioA and AioA-like protein clades identified in Saunders et al. [29] were downloaded from NCBI using the ncbi-genome-download tool (https://github.com/kblin/ncbi-genome-download). Approximately-maximum-likelihood phylogenetic trees were generated using Fasttree [86] specifying 10,000 resamples and using standard settings for everything else. For tree in Fig. 4A, fragmented sequences (shorter than 280aa) were removed. Visualization of resultant trees used the ete3 toolkit [87]. To perform the neighborhood frequency analysis, ten genes upstream and downstream from the *aioA* or *idrA* locus were extracted from the associated GenBank files for each genome, and MMseqs2 was used to cluster homologous proteins as follows [40]: An all- vs. -all search using MMseqs2 was performed using e-value: 0.001, sensitivity: 7.5, and cover: 0.5. A sequence similarity network was built based on the pairwise similarities and the greedy set cover algorithm from MMseqs2 was performed to define protein subclusters as described in detail by Méheust et al. [88]. The resulting subclusters were defined as subfamilies. The cutoff for the IdrA HMM was ultimately determined by iteratively setting the score to a value that excludes genomes that lack the IdrP_1_/IdrP_2_ homologs adjacent to IdrAB and set to a threshold of 640. To search for cld in the downloaded genomes, a profile-HMM for cld, described previously, was used [89]. Frequency was calculated as number of genomes in possession of a cluster divided by the total number of genomes. Projections of this data were drawn using a custom Python 3.7 script. All tanglegram analyses used Dendroscope to load trees for processing and visualization [46].

### Distribution of iodate reductase in ocean metagenomes

The profile-HMM for iodate reductase (described above) was used to search all 40 million nonredundant open reading frames from the 243-sample Tara oceans dataset. Open reading frames were downloaded (available from https://www.ebi.ac.uk/ena/data/view/PRJEB7988) and translated to amino acid sequences using custom BioPython code [90–92]. The amino acid sequences in the 0.22-micron and 0.45-micron range were then searched for hits using the IdrA profile-HMM set at a threshold score of 640. Hits were then grouped by station for further analysis. Reads were mapped to scaffolds with Bowtie2 [93] using the paired end read mapper at default settings and reads were counted using SAMtools [94]. Read abundance mapping to these unique IdrA hits were quantified by using the TPM method for read quantification as described in Ribicic et al. [58, 59]. Ten variables in the metadata associated with the chemical environment at each sampling location were analyzed using the principal component analysis module on scikit-learn 0.23.1 [95]. All sites regardless of *idrA* presence were included in the analysis. Missing metadata values were imputed using the Multivariate Imputation by Chained Equations method (MICE) [96]. Variables included in the analysis were “Sampling depth (m)”, “Mean_Temperature (deg C)”, “Mean_Salinity (PSU)”, “Mean_Oxygen (umol/kg)”, “Mean_Nitrates (umol/L)”, “NO_2_ (umol/L)”, “PO_4_ (umol/L)”, “SI (umol/L)”, “NO_2_NO_3_ (umol/L)”, and irradiance “AMODIS:PAR8d, Einsteins/m-2/d-1”. Components were built using “pca.fit_transform()” and confidence ellipses at one standard deviation were set for each group. Component coefficients were extracted from principal components by using “pca.components_” and displayed as a loadings plot. Explained variance was also extracted from “pca.components_” to display on PCA axes. The map of *idrA* abundance was created using Cartopy 0.17.

## Supplementary information

Supplementary Text

Figure S1

Figure S2

Figure S3

Figure S4

Figure S5

Table S1

Table S2

Table S3
