# Supplementary material for: Genetic and phylogenetic analysis of dissimilatory iodate-reducing bacteria identifies potential niches across the world’s oceans
Source: ISME J. 2021 Jul 2;16(1):38–49. doi: 10.1038/s41396-021-01034-5 (PMC8692401; doi:10.1038/s41396-021-01034-5)
Supplement: Supplementary file 1 — Supplementary Text [file 41396_2021_1034_MOESM1_ESM.docx]

**Supplemental Figures/Tables**

Table S1: Plasmids, strains, and primers used

Table S2: List of proteins shared between denitromonas. sp. IR-12 and P. stutzeri sp. SCT

List of all 26 proteins identified as shared homologs between *Denitromonas* sp. IR-12 and *P. stutzeri* sp. SCT. IR-12 denotes protein in *Denitromonas* sp. IR-12 and SCT denotes protein in *P. stutzeri*. Alternating colors represents a group of homologs. Shared genes belonging to the iodate reduction island are in bold purple. Locus tags for *Denitromonas* sp. IR-12 correspond to Prokka annotation.

Table S3: Select bacteria from figure 4B possessing the IRI and associated environment

Figure S1: Expanded gene neighborhood tree

An expanded tree of the molybdopterin oxidoreductase phylogeny (left) showing a representative subset of genomes identified from Figure 4B. *Denitromonas sp. IR-12* is illustrated in bold. Genome neighborhoods (right) show 10 genes upstream and downstream (if present) from the *idrA* or *aioA* locus. Individual genes were clustered into groups based on amino acid similarity using MMSeqs2 and the frequency of each cluster across all genomes is colored by the intensity of purple where 1.0 means the cluster is present in all genomes, and 0.0 means the cluster is absent in all genomes. Circles above each gene represents the gene cluster protein family. Molybdopterin oxidoreductase (●), the associated Rieske containing subunit (●), or the di-haem cytochrome c peroxidases (●) are labeled.

Figure S2: Tanglegram analysis of individual proteins in the IRI

A pairwise tanglegram analysis between IdrA (A), IdrB (B), and IdrP_1_/P_2_ (C), and Rps3. Labels for each tree have been removed for ease of viewing. Rps3 trees in all figures use *Halobiforma lacisalsi* as an outgroup, which is removed when corresponding IRI gene is not present in the organism (C). Lines drawn between trees represent a connection between the physical location on the left tree and the physical connection on the right tree. Entanglement in A-C demonstrates the discordant tree topology, and is suggestive of horizontal gene transfer. A comparison between IdrA and IdrB (D) is shown to demonstrate the co-evolution of these two proteins together.

Figure S3: Individual TPM counts at Tara stations and correlation to unique idrA genes

Chart on the left shows the TPM of individual hits on a scaffold organized by Tara location identifier. Coloration represents the individual Tara station while marker shape indicates general geographic location. Chart on the right correlates the number of unique IdrA hits at any given site to the cumulative TPM at an individual location. Tara station is denoted by color and general geographic location is denoted by marker shape.

Figure S4: Cumulative TPM plotted by depth and colored by concentration

All three charts demonstrate the cumulative TPM at an individual Tara station plotted against sampling depth. Stations with no IdrA hits are omitted. Color corresponds to the concentration of oxygen (blues), phosphates (oranges), or nitrates (greens). Darker colors represent higher concentrations. Color scales are normalized to the maximum concentration for each variable within the IdrA+ dataset.

Figure S5: Loading plot for PCA

A loading plot of the ten variables used in the first two principal components. Variables are identified at the end of each arrow.

**Supplemental Methods**

Expression vector transformation

Electroporator model GenePulser XCell (BioRad, USA) was used for high efficiency transformation of expression vectors into *Denitromonas* sp. IR-12. The procedure in short: 40 mL of *Denitromonas* sp. IR-12 was grown for 72 hours and spun down at 4000 RPM at 4°C for 15 minutes in 6 mL of cold, sterile deionized H_2_O, followed by a wash and spin down in 6mL of cold, sterile 10% glycerol for an additional 15 minutes. The supernatant is decanted, and the pellet is resuspended to a final volume of 600 µL of 10% glycerol. Purified plasmid DNA is added at a concentration of 10ng/µL, and cells sit on ice for a minimum of 10 minutes. Electroporator is then pulsed once at 1750V, 25µF, and 400Ω for a 1mm GenePulser cuvette (BioRad, USA). Cells are then transferred to 500 µL of R2A media and are recovered for 4 hours at 30°C. After outgrowth 100µL of cells are plated out on R2A plates with 5 µg/µL kanamycin and allowed to grow at 30°C for three days. A 1:10 dilution is recommended due to high transformation efficiency. Since the concentration of kanamycin is relatively low, all colonies are screened for the presence of the kanamycin resistance marker via PCR.

Suicide vector transformation

WM3064 *E. coli* was used to efficiently deliver suicide vectors into *Denitromonas* sp. IR-12. 50mL of *Denitromonas* sp. IR-12 is grown aerobically in R2A at 30°C for 30 hours, and *E. coli* is grown from an overnight culture for 4-6 hours at 37°C. Cultures are harvested and spun down at 7000 RCF at room temperature for 5 minutes. Cultures are then washed in R2A containing diaminopimelic acid (DAP) and are separately resuspended to a final volume of 250µL in R2A+DAP. The optical densities of each culture are measured, and the cells are added together and mixed at a 1:1 ratio in a final volume of 500µL. The entire conjugation is spotted onto three separate R2A+DAP plates and incubated at 30°C for 24 hours. The entire conjugation is then scraped off and resuspended in 10mL of R2A (no DAP) with 15% glycerol. 100µL of the conjugation is then spread out on two separate R2A plates with kanamycin and allowed to grow for 5 days. Transconjugants are then picked and plated again on R2A plates with kanamycin to allow for additional growth. Transconjugants are then screened by PCR for plasmid integration, and transconjugants possessing the rare integration event are picked and grown in liquid R2A for 3 days. 100µL of selected transconjugants are plated out on R2A + 7.5% sucrose, and colonies are picked and screened on R2A + 7.5% sucrose and R2A + kanamycin after 5 days. Colonies showing growth on R2A + sucrose and no growth on R2A + kanamycin are then screened for the removal of the suicide vector and the presence of the expected deletion.

Vitamin and Mineral Mixes

Vitamin and the two mineral mixes were prepared separately as stock solutions. Per liter the vitamin mix contains, 2.0 mg D-biotin, 2.0 mg folic acid, 10.0 mg pyroxidine HCl, 5.0 mg riboflavin, 5.0 mg thiamine, 5.0 mg nicotinic acid, 5.0 mg pantothenic acid, 0.1 mg vitamin B12, 5.0 mg p-amino benzoic acid, 5.0 mg D,L-6,8-thiotic acid. Per liter mineral mix one contains 1.5 mg NTA disodium salt, 3.0 mg MgSO_4_·7H2O, 0.5 mg MnSO_4_·H2O, 1.0 mg NaCl, 0.1 mg FeSO_4_·7H2O, 0.1 mg CaCl_2_·2H2O, 0.1 mg CoCl_2_·6H2O, 0.13 mg ZnCl, 0.1 mg CuSO_4_·5H2O, 0.1 mg AlK(SO_4_)_2_·12H2O, 0.1 mg Boric Acid, 0.025 mg Na_2_MoO_4_·2H2O, 0.024 mg NiCl_2_·6H2O, 0.025 mg Na_2_WO_4_·2H2O, 0.02 mg Na_2_SeO_4_. Mineral mix one is then adjusted to a pH of 6.0 with 1M NaOH. Per liter mineral mix two contains 40.0 g NaCl, 50.0g NH_4_Cl, 5.0 g KCl, 5.0 g KH_2_PO_4_, 10.0g MgSO_4_·7H2O, and 1.0g CaCl_2_·2H2O. Mineral mix two is then adjusted to pH 6.0 with KOH.

Media with vitamin and mineral mixes, add all of the above mixes. Per liter of media, 10 mL of the vitamin mix, 10 mL of the mineral mix one, and 20 mL of mineral mix two are added.

**References for Table S3**

1. Chao LS-L, Davis RE, Moyer CL. Characterization of bacterial community structure in vestimentiferan tubeworm *Ridgeia piscesae* trophosomes. Mar Ecol. 2007;28(1):72-85.

2. Patra AK, Cho HH, Kwon YM, Kwon KK, Sato T, Kato C, et al. Phylogenetic relationship between symbionts of tubeworm *Lamellibrachia satsuma* and the sediment microbial community in Kagoshima Bay. Ocean Sci J. 2016;51(3):317-332.

3. Gardebrecht A, Markert S, Sievert SM, Felbeck H, Thürmer A, Albrecht D, et al. Physiological homogeneity among the endosymbionts of *Riftia pachyptila* and *Tevnia jerichonana* revealed by proteogenomics. ISME J. 2012;6(4):766-776.

4. Wang Z, Zhang Z, Hu Z, Zhao J, Zhao D, Zhang Y. *Alginatibacterium sediminis* gen. nov., sp. nov., a novel marine gammaproteobacterium isolated from coastal sediment. Int J Syst Evol Microbiol. 2019;69(2):511-516.

5. Park S, Park J-M, Kang C-H, Yoon J-H. *Aliiroseovarius pelagivivens* gen. nov., sp. nov., isolated from seawater, and reclassification of three species of the genus *Roseovarius* as *Aliiroseovarius crassostreae* comb. nov., *Aliiroseovarius halocynthiae* comb. nov. and *Aliiroseovarius sediminilitoris* comb. nov. Int J Syst Evol Microbiol. 2015;65(Pt_8):2646-2652.

6. Kasai Y, Takahata Y, Manefield M, Watanabe K. RNA-based stable isotope probing and isolation of anaerobic benzene-degrading bacteria from gasoline-contaminated groundwater. Appl Environ Microbiol. 2006;72(5):3586-3592.

7. Jung Y-T, Park S, Lee J-S, Yoon J-H. *Defluviimonas aquaemixtae* sp. nov., isolated from the junction between a freshwater spring and the ocean. Int J Syst Evol Microbiol. 2014;64(Pt_12):4191-4197.

8. Jin HM, Lee HJ, Kim JM, Park MS, Lee K, Jeon CO. *Litorimicrobium taeanense* gen. nov., sp. nov., isolated from a sandy beach. Int J Syst Evol Microbiol. 2011;61(6):1392-1396.

9. Zhang D-C, Li H-R, Xin Y-H, Chi Z-M, Zhou P-J, Yu Y. *Marinobacter psychrophilus* sp. nov., a psychrophilic bacterium isolated from the Arctic. Int J Syst Evol Microbiol. 2008;58(6):1463-1466.

10. Kim HJ, Park S, Lee JM, Park S, Jung W, Kang J-S, et al. *Moritella dasanensis* sp. nov., a psychrophilic bacterium isolated from the Arctic ocean. Int J Syst Evol Microbiol. 2008;58(4):817-820.

11. Nogi Y, Kato C. Taxonomic studies of extremely barophilic bacteria isolated from the Mariana Trench and description of *Moritella yayanosii* sp. nov., a new barophilic bacterial isolate. Extremophiles. 1999;3(1):71-77.

12. Hameed A, Shahina M, Lai W-A, Lin S-Y, Young L-S, Liu Y-C, et al. *Oricola cellulosilytica* gen. nov., sp. nov., a cellulose-degrading bacterium of the family *Phyllobacteriaceae* isolated from surface seashore water, and emended descriptions of *Mesorhizobium loti* and *Phyllobacteriummyrsinacearum*. Antonie Van Leeuwenhoek. 2015;107(3):759-771.

13. Hahnke S, Tindall BJ, Schumann P, Simon M, Brinkhoff T. *Pelagimonas varians* gen. nov., sp. nov., isolated from the southern North Sea. Int J Syst Evol Microbiol. 2013;63(Pt_3):835-843.

14. Li Y, Zhou M, Wang F, Wang ET, Du Z, Wu C, et al. *Photobacterium proteolyticum* sp. nov., a protease-producing bacterium isolated from ocean sediments of Laizhou Bay. Int J Syst Evol Microbiol. 2017;67(6):1835-1840.

15. Amachi S, Kawaguchi N, Muramatsu Y, Tsuchiya S, Watanabe Y, Shinoyama H, et al. Dissimilatory iodate reduction by marine *Pseudomonas* sp. strain SCT. Appl Environ Microbiol. 2007;73(18):5725-5730.

16. Rajasabapathy R, Mohandass C, Dastager SG, Liu Q, Khieu T-N, Son CK, et al. *Roseovarius azorensis* sp. nov., isolated from seawater at Espalamaca, Azores. Antonie Van Leeuwenhoek. 2014;105(3):571-578.

17. Jones DS, Bailey JV, Flood BE. *Sedimenticola thiotaurini* sp. nov., a sulfur-oxidizing bacterium isolated from salt marsh sediments, and emended descriptions of the genus *Sedimenticola* and *Sedimenticola selenatireducens*. Int J Syst Evol Microbiol. 2015;65(8):2522-2530.

18. Alex A, Antunes A. Comparative Genomics Reveals Metabolic Specificity of *Endozoicomonas* isolated from a marine sponge and the genomic repertoire for host-bacteria symbioses. Microorganisms. 2019;7(12):635.

19. Pujalte MJ, Macián MC, Arahal DR, Garay E. *Stappia alba* sp. nov., isolated from Mediterranean oysters. Syst Appl Microbiol. 2005;28(8):672-678.

20. Kim Y-O, Park S, Nam B-H, Park J-M, Kim D-G, Yoon J-H. *Litoreibacter ascidiaceicola* sp. nov., isolated from the golden sea squirt *Halocynthiaaurantium*. Int J Syst Evol Microbiol. 2014;64(Pt_8):2545-2550.

21. Colquhoun D, Hovland H, Hellberg H, Haug T, Nilsen H. *Moritella viscosa* isolated from farmed Atlantic cod (Gadus morhua). Bull Eur Assoc Fish Pathol. 2004;24(2):109-114.

22. Huang Z, Guo F, Lai Q, Shao Z. *Notoacmeibacter marinus* gen. nov., sp. nov., isolated from the gut of a limpet and proposal of *Notoacmeibacteraceae* fam. nov. in the order *Rhizobiales* of the class *Alphaproteobacteria*. Int J Syst Evol Microbiol. 2017;67(8):2527-2531.

23. Yan X-R, Tuo L. *Notoacmeibacter ruber* sp. nov., a novel endophytic bacterium isolated from leaf of *Rhizophora stylosa*. Antonie Van Leeuwenhoek. 2019;112(6):919-925.

24. O’Halloran JA, Barbosa TM, Morrissey JP, Kennedy J, Dobson ADW, O’Gara F. *Pseudovibrio axinellae* sp. nov., isolated from an Irish marine sponge. Int J Syst Evol Microbiol. 2013;63(Pt_1):141-145.

25. Jung HS, Jeong SE, Chun BH, Quan Z-X, Jeon CO. *Rhodophyticola porphyridii* gen. nov., sp. nov., isolated from a red alga, Porphyridium marinum. Int J Syst Evol Microbiol. 2019;69(6):1656-1661.

26. Shiba T. *Roseobacter litoralis* gen. nov., sp. nov., and *Roseobacter denitrificans* sp. nov., aerobic pink-pigmented bacteria which contain bacteriochlorophyll a. Syst Appl Microbiol. 1991;14(2):140-145.

27. Kim Y-O, Park S, Nam B-H, Kang S-J, Hur YB, Lee S-J, et al. *Ruegeria halocynthiae* sp. nov., isolated from the sea squirt Halocynthia roretzi. Int J Syst Evol Microbiol. 2012;62(Pt_4):925-930.

28. Flood BE, Fliss P, Jones DS, Dick GJ, Jain S, Kaster A-K, et al. Single-cell (meta-)genomics of a dimorphic candidatus *Thiomargarita nelsonii* reveals genomic plasticity. Front Microbiol. 2016;7(603).

29. Beaz-Hidalgo R, Doce A, Pascual J, Toranzo AE, Romalde JL. *Vibrio gallaecicus* sp. nov. isolated from cultured clams in north-western Spain. Syst Appl Microbiol. 2009;32(2):111-117.

30. Gomez-Gil B, Fajer-Avila E, Pascual J, Macián MC, Pujalte MJ, Garay E, et al. *Vibrio sinaloensis* sp. nov., isolated from the spotted rose snapper, *Lutjanus guttatus* Steindachner, 1869. Int J Syst Evol Microbiol. 2008;58(7):1621-1624.

31. Gómez-León J, Villamil L, Lemos ML, Novoa B, Figueras A. Isolation of *Vibrio alginolyticus* and *Vibrio splendidus* from aquacultured carpet shell clam *Ruditapes decussatus* larvae associated with mass mortalities. Appl Environ Microbiol. 2005;71(1):98-104.

32. Borrego JJ, Castro D, Luque A, Paillard C, Maes P, Garcia MT, et al*. Vibrio tapetis* sp. nov., the causative agent of the brown ring disease affecting cultured clams. Int J Syst Evol Microbiol. 1996;46(2):480-484.

33. Thompson FL, Thompson CC, Swings J. *Vibrio tasmaniensis* sp. nov., isolated from Atlantic Salmon (Salmo salar L.). Syst Appl Microbiol. 2003;26(1):65-69.

34. Kojima H, Watanabe M, Fukui M. *Sulfurivermis fontis* gen. nov., sp. nov., a sulfur-oxidizing autotroph, and proposal of *Thioprofundaceae* fam. nov. Int J Syst Evol Microbiol. 2017;67(9):3458-3461.
