# Supplementary figures and images for: Genetic and phylogenetic analysis of dissimilatory iodate-reducing bacteria identifies potential niches across the world’s oceans
Source: ISME J. 2021 Jul 2;16(1):38–49. doi: 10.1038/s41396-021-01034-5 (PMC8692401; doi:10.1038/s41396-021-01034-5)

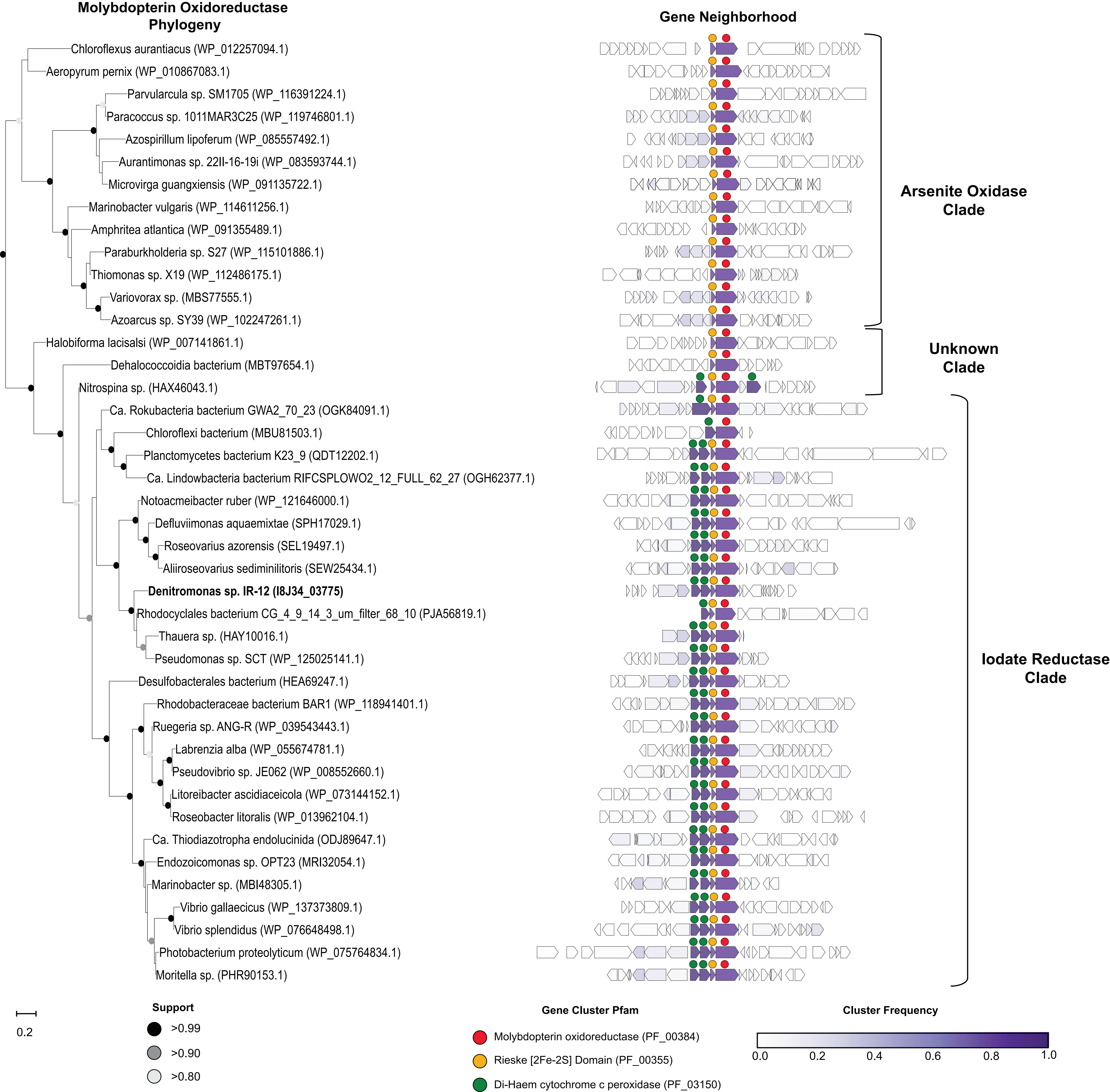

Supplement: Supplementary file 2 — Figure S1 [file 41396_2021_1034_MOESM2_ESM.pdf]

A

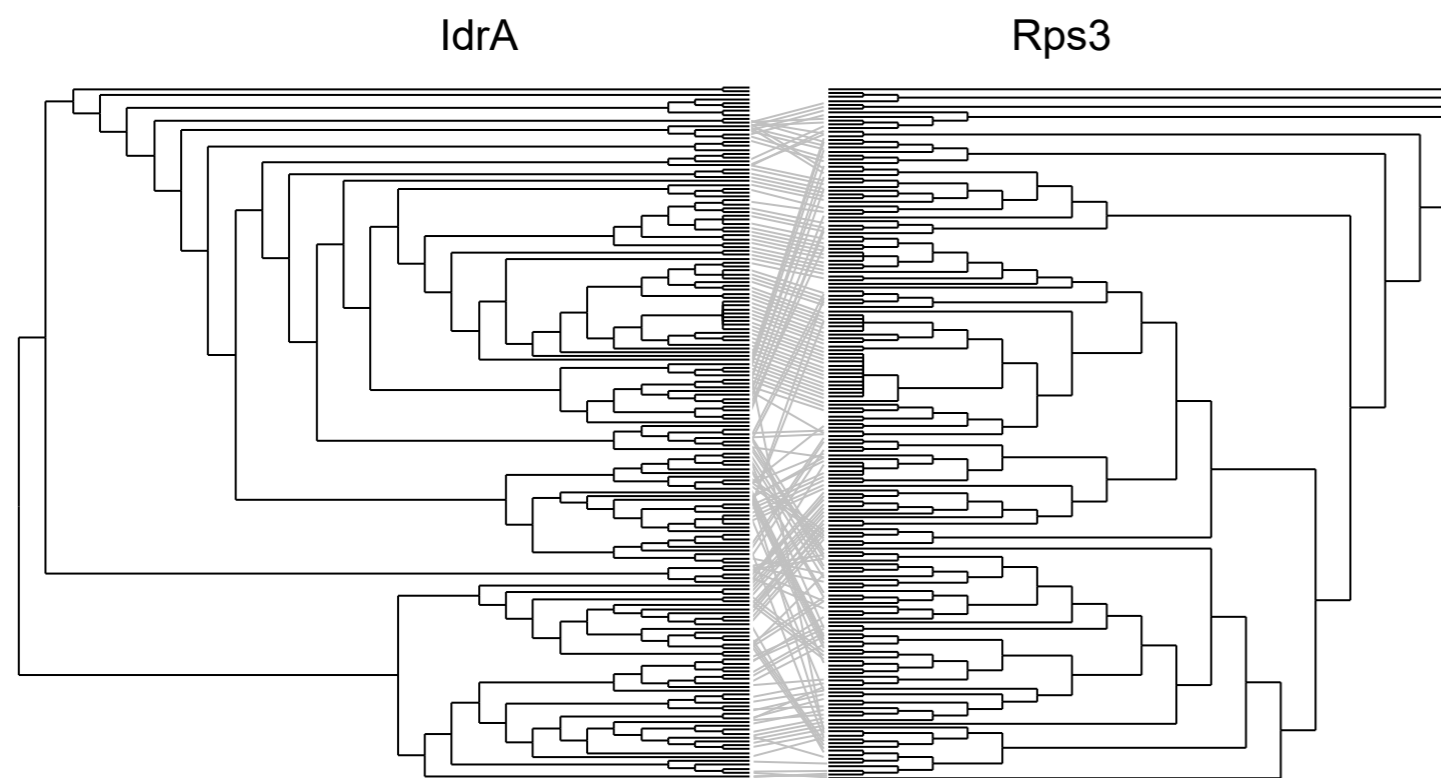

B

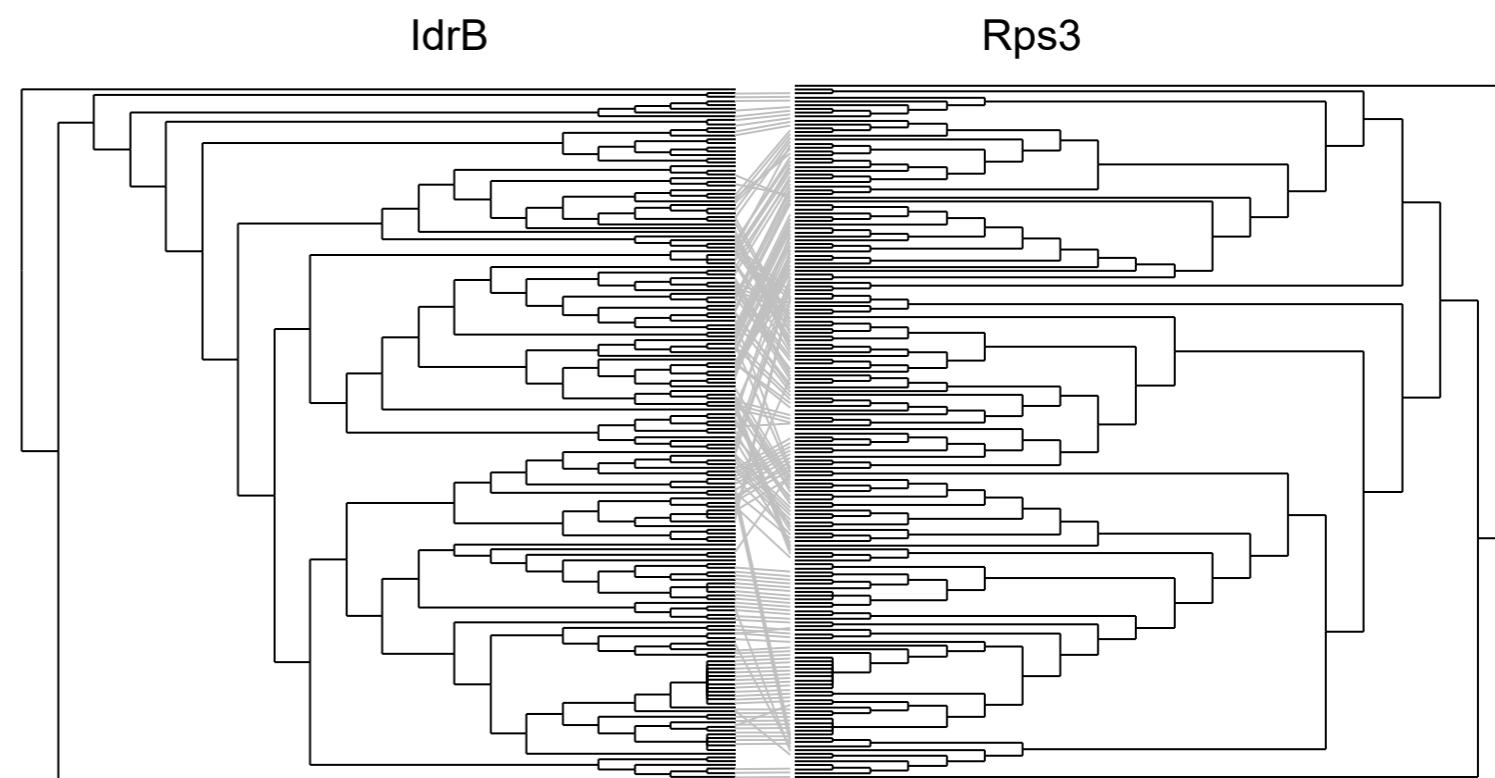

C

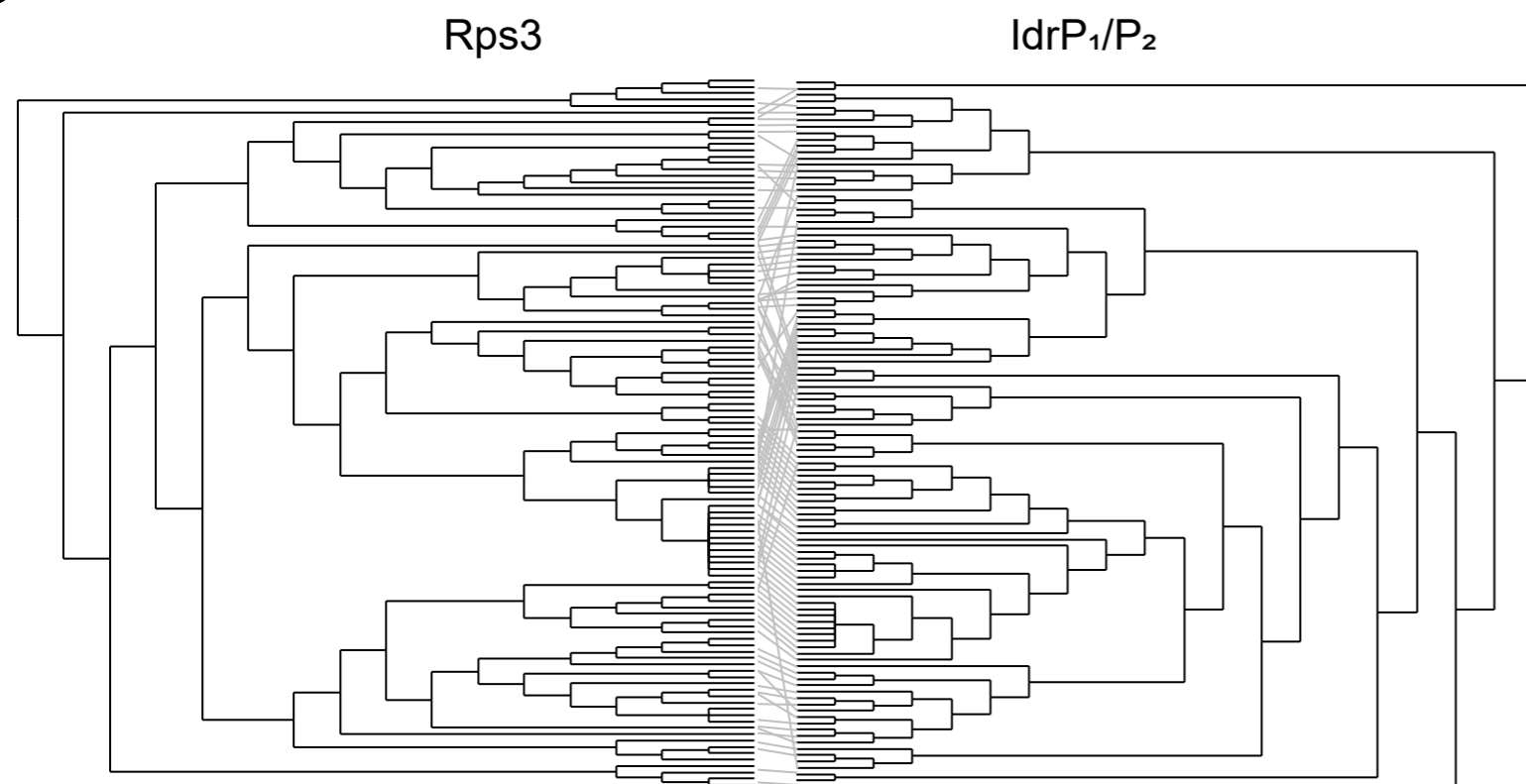

D

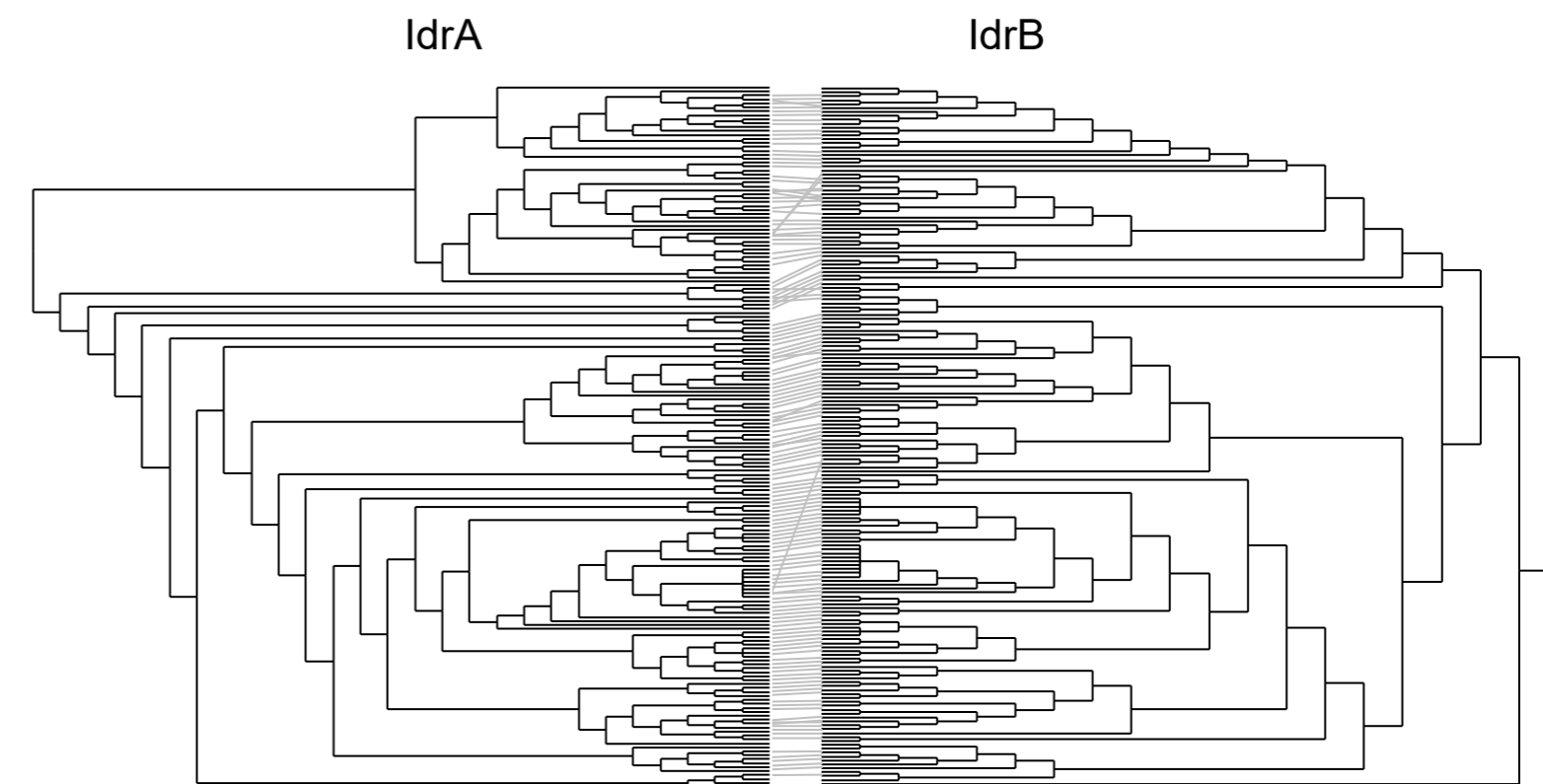

Supplement: Supplementary file 3 — Figure S2 [file 41396_2021_1034_MOESM3_ESM.pdf]

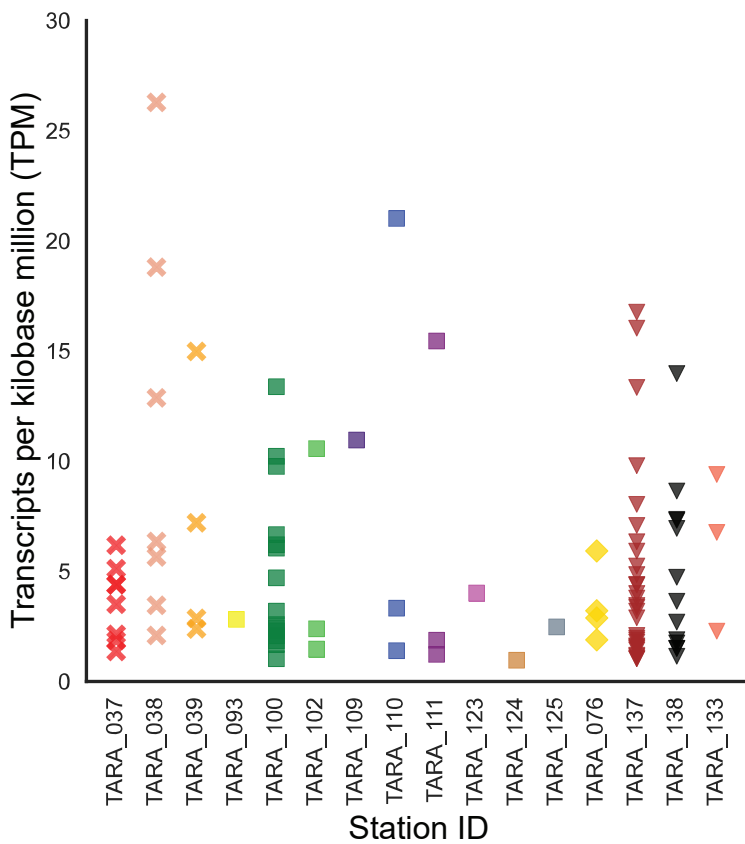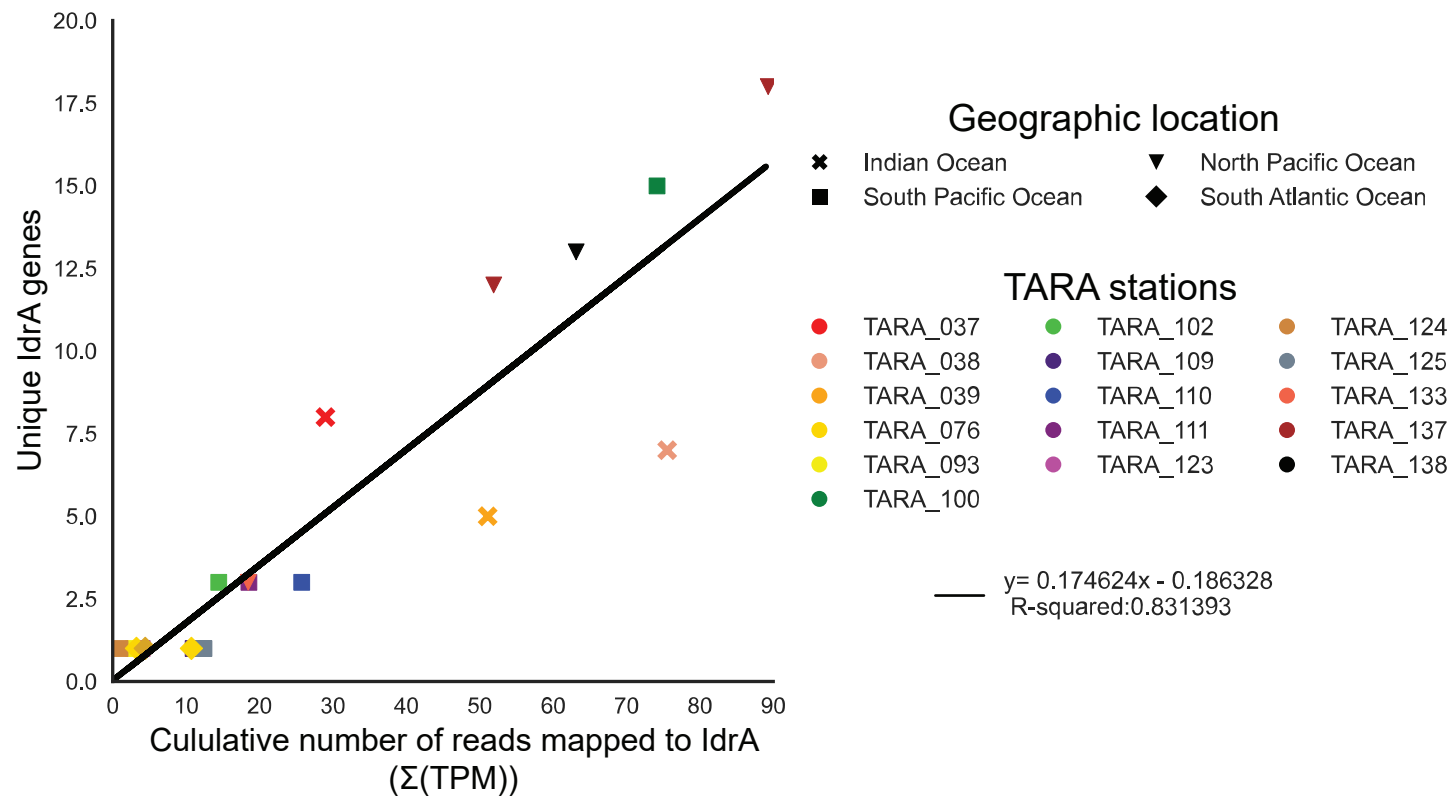

Supplement: Supplementary file 4 — Figure S3 [file 41396_2021_1034_MOESM4_ESM.pdf]

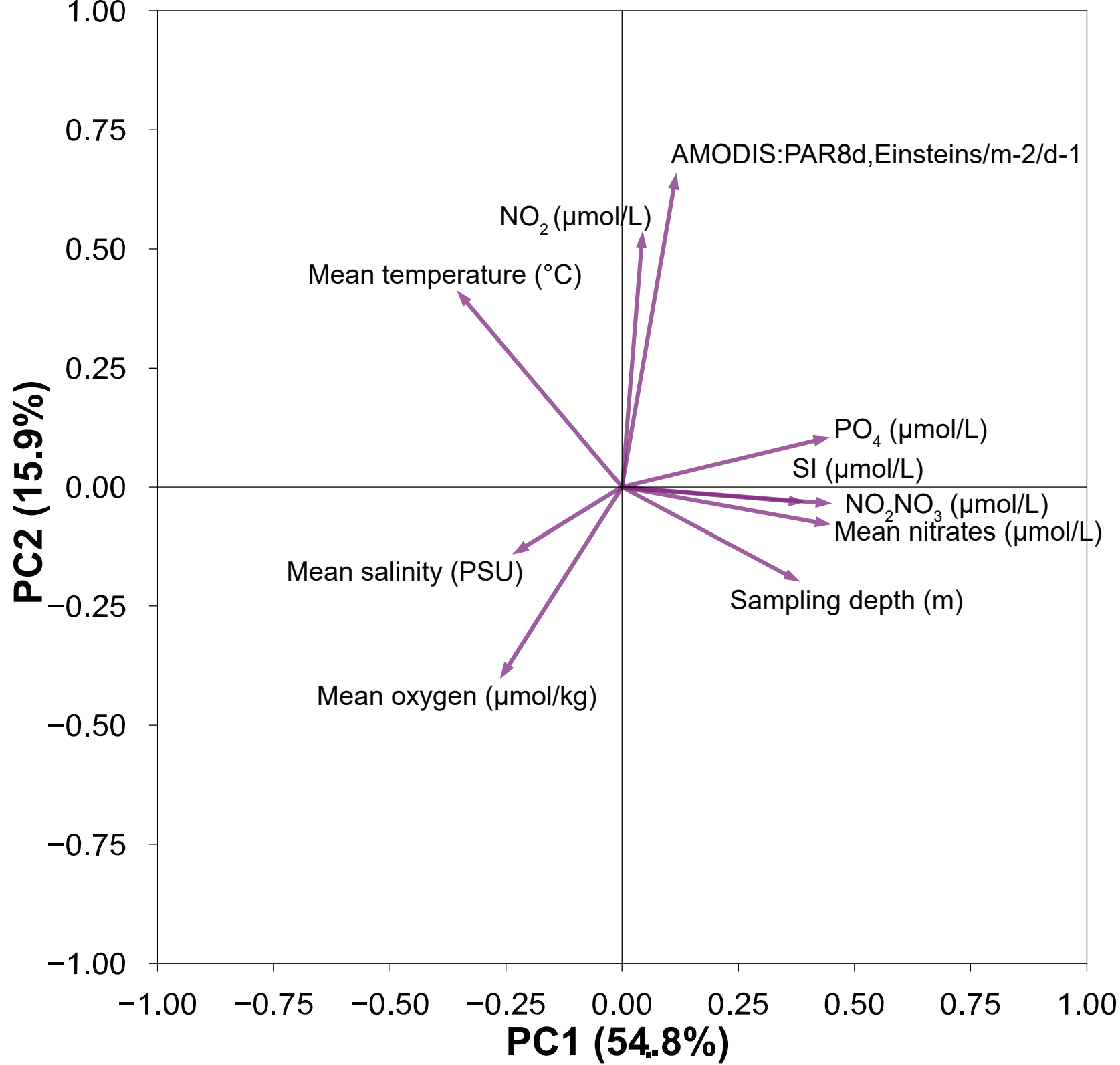

Supplement: Supplementary file 6 — Figure S5 [file 41396_2021_1034_MOESM6_ESM.pdf]
