# Supplementary material for: Genetic and phylogenetic analysis of dissimilatory iodate-reducing bacteria identifies potential niches across the world’s oceans
Source: ISME J. 2021 Jul 2;16(1):38–49. doi: 10.1038/s41396-021-01034-5 (PMC8692401; doi:10.1038/s41396-021-01034-5)
Supplement: Supplementary file 5 — Figure S4 [file 41396_2021_1034_MOESM5_ESM.pdf]

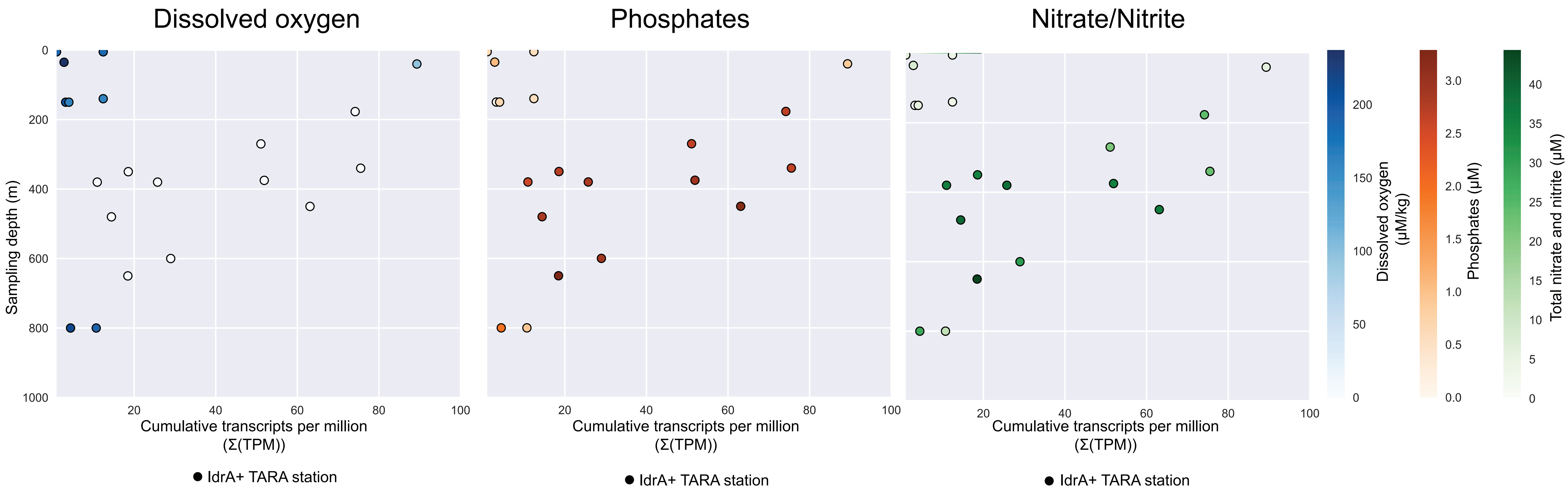
Dissolved oxygen
Phosphates
Nitrate/Nitrite
Sampling depth (m)
0
200
400
600
800
1000
20
40
60
80
100
Cumulative transcripts per million
(Σ(TPM))
● IdrA+ TARA station
Dissolved oxygen
(µM/kg)
Phosphates (µM)
Total nitrate and nitrite (µM)
